# Comprehensive de novo mutation discovery with HiFi long-read sequencing

**DOI:** 10.1186/s13073-023-01183-6

**Published:** 2023-05-08

**Authors:** Erdi Kucuk, Bart P. G. H. van der Sanden, Luke O’Gorman, Michael Kwint, Ronny Derks, Aaron M. Wenger, Christine Lambert, Shreyasee Chakraborty, Primo Baybayan, William J. Rowell, Han G. Brunner, Lisenka E. L. M. Vissers, Alexander Hoischen, Christian Gilissen

**Affiliations:** 1 Department of Human Genetics, Radboud University Medical Center, PO Box 9101, 6500 HB Nijmegen, The Netherlands; 2 Radboud Institute for Molecular Life Sciences, Radboud University Medical Center, Nijmegen, The Netherlands; 3 Donders Institute for Brain, Cognition and Behaviour, Radboud University Medical Center, Nijmegen, The Netherlands; 4 Pacific Biosciences, Menlo Park, CA USA; 5 Department of Clinical Genetics, Maastricht University Medical Center, Maastricht, The Netherlands; 6 GROW School for Oncology and Developmental Biology, Maastricht University Medical Center, Maastricht, The Netherlands; 7 Department of Internal Medicine, Radboud University Medical Center for Infectious Diseases (RCI), Radboud University Medical Center, Nijmegen, the Netherlands

**Keywords:** Long-read sequencing, HiFi reads, De novo mutations

## Abstract

**Background:**

Long-read sequencing (LRS) techniques have been very successful in identifying structural variants (SVs). However, the high error rate of LRS made the detection of small variants (substitutions and short indels < 20 bp) more challenging. The introduction of PacBio HiFi sequencing makes LRS also suited for detecting small variation. Here we evaluate the ability of HiFi reads to detect de novo mutations (DNMs) of all types, which are technically challenging variant types and a major cause of sporadic, severe, early-onset disease.

**Methods:**

We sequenced the genomes of eight parent–child trios using high coverage PacBio HiFi LRS (~ 30-fold coverage) and Illumina short-read sequencing (SRS) (~ 50-fold coverage). De novo substitutions, small indels, short tandem repeats (STRs) and SVs were called in both datasets and compared to each other to assess the accuracy of HiFi LRS. In addition, we determined the parent-of-origin of the small DNMs using phasing.

**Results:**

We identified a total of 672 and 859 de novo substitutions/indels, 28 and 126 de novo STRs, and 24 and 1 de novo SVs in LRS and SRS respectively. For the small variants, there was a 92 and 85% concordance between the platforms. For the STRs and SVs, the concordance was 3.6 and 0.8%, and 4 and 100% respectively. We successfully validated 27/54 LRS-unique small variants, of which 11 (41%) were confirmed as true de novo events. For the SRS-unique small variants, we validated 42/133 DNMs and 8 (19%) were confirmed as true de novo event. Validation of 18 LRS-unique de novo STR calls confirmed none of the repeat expansions as true DNM. Confirmation of the 23 LRS-unique SVs was possible for 19 candidate SVs of which 10 (52.6%) were true de novo events. Furthermore, we were able to assign 96% of DNMs to their parental allele with LRS data, as opposed to just 20% with SRS data.

**Conclusions:**

HiFi LRS can now produce the most comprehensive variant dataset obtainable by a single technology in a single laboratory, allowing accurate calling of substitutions, indels, STRs and SVs. The accuracy even allows sensitive calling of DNMs on all variant levels, and also allows for phasing, which helps to distinguish true positive from false positive DNMs.

**Supplementary Information:**

The online version contains supplementary material available at 10.1186/s13073-023-01183-6.

## Background

A comprehensive characterization of variation of individual human genomes is of great importance to gain insight into genetic traits and diseases [1]. For rare disease studies, it is especially important to identify the full spectrum of all variant types, including substitutions, indels, short tandem repeats (STRs) and structural variants (SVs). A particular challenge for the accuracy of genomic technologies are de novo mutations (DNMs) [2, 3], which have been shown to be a major cause of sporadic, severe, early-onset disease [3, 4]. DNMs are mutations that arise in the germline of one of the parents during gamete formation and are transmitted to the offspring. Every human genome contains roughly between 40 and 90 DNMs on average [3]. They are however also among the most challenging variants to identify, as DNM call sets typically contain large number of false positive calls due to sequencing artifacts, mapping artifacts, differences in sequence coverage and mosaicism [4–8]. Therefore, comprehensive detection of DNMs of all types demands the highest-quality sequencing data.

Whereas short-read sequencing (SRS) can be used to accurately call small variants, such as substitutions and small indels (< 20 bp), the sensitivity to detect large STRs, copy number variants (CNVs), and SVs is limited as this can only be done by inference from systematic deviations in read coverage or read alignments [9]. Long-read sequencing (LRS) technologies typically generate sequencing reads of 10 to 100 kilo bases (kb) in size which offers many advantages compared to short-read sequencing [10]. Long reads can interrogate regions of the human genome that are inaccessible by SRS and can encompass complete SV events thereby improving their detection [11, 12]. LRS has therefore been used extensively for de novo assembly of human genomes and for the characterization of structural genome variation that remains undetected by SRS [4, 10, 13, 14]. However, LRS technologies have traditionally suffered from low accuracy at single base pair (bp) resolution, with a raw error rate of 8 to 15%, which did not allow them to reliably detect variants smaller than 50 bp. This reduced accuracy conserves the need to combine LRS with SRS to accurately detect the entire spectrum of de novo variation, which is accompanied by additional costs and time [11].

With improvements in LRS technology and specifically the recent introduction of PacBio HiFi reads, it is now possible to obtain high base call accuracy. With HiFi sequence reads, DNA templates of 10–30 kb in length are subjected to circular consensus sequencing (CCS) allowing to derive a consensus sequence of both strands of the insert region from multiple passes of the polymerase over a single template molecule [11, 15, 16]. The number of passes determines the accuracy of the consensus reads, since each pass allows for better error correction in the consensus sequence. HiFi reads are defined as reads with an accuracy of at least 99% (Phred quality score 20), theoretically resulting in the detection of substitutions and small indels being on par with SRS technology [17]. HiFi technology has already been used to identify SVs in patients suffering from different genetic disorders, including synpolydactyly, syndromic intellectual disability, choroideremia, and teratoid rhabdoid tumors [18–21]. The increased base accuracy of HiFi sequencing should be especially advantageous for the detection of small DNMs and could even allow for improved sensitivity compared to SRS. Here, we investigated whether HiFi sequencing is sufficiently accurate to allow for the comprehensive detection of all types of de novo variation in parent–child trio genomes, which would remove the necessity to complement LRS by SRS and result in most comprehensive genomes.

## Methods

### Patient selection

The department of Human Genetics of the Radboudumc is a tertiary referral center for patients with neurodevelopmental disorder (NDD) in the Netherlands. For this study, we selected 8 index patients with an NDD of suspected genetic origin and their unaffected parents (Additional file 1: Table S1) from a previous study in which a total of 150 NDD patient-parent trios were prospectively included between October 1st 2018 and July 1st 2019 [22]. The only two inclusion criteria for the current study were that the clinical geneticist requested a genetic diagnostic test to identify the molecular defect underlying the patient’s phenotype and that no disease-causing variant was identified by both whole exome sequencing and short-read whole genome sequencing [23]. Board-certified clinical geneticists counseled all 8 patients and their parents for the SRS procedures. All participants or their legal representatives gave written informed consent. This study was approved by the Medical Review Ethics Committee Oost-Nederland and Radboudumc Institutional Review Board under 2020–6853, as part of 2018–4985 and 2014–1254.

### Long-read sequencing and variant calling

For the LRS, we targeted 30 × HiFi coverage by using at least 3 SMRT Cells per sample (Additional file 1: Table S1). All samples were processed in the same fashion, according to the manufacturer’s instructions (PacBio, Menlo Park, CA, USA). In brief, 5 µg DNA was sheared on Megaruptor 3 (Diagenode, Liège, Belgium) to a target size of 18 kb, libraries were prepared with SMRTbell express template prep kit 2.0 (PacBio, Menlo Park, CA, USA), size-selected > 10 kb on the PippinHT (Sage Science, Beverly, MA, USA), and sequenced for 30 h on the Sequel II system using Chemistry 2.0. HiFi reads were generated with CCS 4.2.0 and then processed using our in-house script which is available on Github [24].

Sequencing reads were aligned to the GRCh38/Hg38 genome with pbmm2 (version 1.4.0) [17, 25], using default parameters. Small variant (substitution and indel) calling was performed using DeepVariant (version 1.1.0) with default settings [26]. No threshold for maximum size of the indels was applied, and all indel calls were used for further analyses. STR calling was performed using Tandem Repeat Genotyper (TRGT; version 0.3.3) at 171,146 highly polymorphic repeat loci that are described in a tandem repeat catalog that is available together with the TRGT tool [27]. SV calling was performed using PBSV (version 2.4.0) [17] default settings with a minimum SV size of 20 bp.

From the total variant call set, we filtered de novo mutations (i.e., substitutions, indels, and structural variants; DNMs) using slivar [28] (0.2.7) with two different sets of filter criteria. For the SVs, we only applied the strict filtering criteria.

For the LRS strict and lenient lists we applied the following filters.

**Table:** 

Parameter	Strict filtering	Lenient filtering
Proband genotype	0/1	0/1
Parental genotype	0/0	0/0
Parental alternative allele depth	0	< 2 total
Proband allele depth	> 5	NA
Reference allele depth	> 10	NA
Total depth	< 50	NA
Quality score	> 30	NA
Genotype quality	> 20	> 10
Allele count in gnomAD and HPRC	< 5	< 5

For the STRs, the output files were first filtered for loci for which all family members had both alleles genotyped. Subsequently, de novo STR expansions and contractions were selected using the number of repeat units of the two genotyped alleles. When the number of repeat units in one or both alleles of the patient was ≥ 2 repeat units longer or shorter than both parents, the repeat locus was considered de novo. Subsequently, we excluded de novo STR calls that were present in more than one patient of this cohort. Additionally, the repeat length had to be an outlier when compared to the alleles of all 23 other samples using the 1.5*interquartile range (IQR) rule. Finally, we excluded the de novo STR calls where one or both alleles had a TRGT quality score ≤ 0.8 (LRS).

### Short-read sequencing and variant calling

Short-read WGS was performed as described by the manufacturer (Illumina, San Diego, CA, USA), and in detail reported in van der Sanden et al. [22]. In brief, 1 µg DNA, isolated from whole blood, was used for library preparation using the Illumina TruSeq DNA PCR-free protocol, with an average insert size of 450 bp. To allow pooling of samples, barcoded indexing was included in the library preparation. Samples were pooled equimolarly on an S2 or S4 flowcell, prior to sequencing on an Illumina NovaSeq instrument to an anticipated genome-wide coverage of 50-fold, with a minimum of 45-fold.

After sequencing, FASTQ files were processed through our in-house pipeline for short-read genomes. Reads were mapped to the human reference genome (GRCh38/Hg38) using BWA (v.0.78) [29] and the quality of the resulting BAM file was assessed using Qualimap [30] (v.2.2.1). Variant calling was performed using various tools to optimize sensitivity per variant type. For calling small variants (substitutions and indels), GATK [31] was used and no threshold for maximum size of the indels was applied. SVs were called using Manta Structural Variant Caller (v.1.1.0; Illumina) [32], following a paired-end and split-read approach for SVs identification. No minimal size threshold was applied. CNVs were called using Canvas Copy Number Variant Caller (v.1.40.0; Illumina) [33] using default parameters. STRs were called using ExpansionHunter [34] using the same tandem repeat catalog containing 171,146 loci as for LRS. De novo STRs were selected the same way as with LRS, except for the last step where low-quality calls were excluded. For SRS, this step was replaced by excluding STR calls where the repeat length of one or both alleles was outside the confidence intervals of ExpansionHunter.

For SRS, we used two independent de novo callers, namely an in-house developed method based on Samtools mpileups and DeNovoCNN [35]. For the in-house method, we first discarded all inherited variants and variants with a gnomAD allele frequency > 0.1% or GATK score < 50. Remaining variants were run through the de novo caller which annotated the variants as inherited or possible de novo based on the Samtools mpileups. Subsequently, we only selected variants with ≥ 20% alternative allele depth, ≥ 5 alternative reads, and ≤ 1% in-house allele frequency. For DeNovoCNN, inherited variants were also discarded, and the tool was run on the remainder of the variant list using default parameters, resulting in variants with a DeNovoCNN probability score > 0.5. Variants called by both de novo callers were considered a true SRS DNM (SRS list). Variants only called by one de novo caller were listed separately (i.e., in-house unique list and DeNovoCNN unique list).

### Variant annotation

Small variants from both LRS and SRS were annotated by an in-house pipeline. This variant annotation was performed using the Variant Effect Predictor (VEP V.91) [36] and Gencode V.34 basic [37] gene annotations. Frequency information was added from GnomAD V.2.1.1 [38] and from an in-house database. In-house gene panel information was added for those genetic variants within a known disease gene.

SVs and CNVs were annotated using an in-house developed pipeline. This pipeline was based on ANNOVAR [39] and Gencode V.34 basic gene annotations. Additional frequency information was added from GnomAD V.2.1 [38], 1000G V.8 [40], and GoNL [41] SV database.

### Comparison of inherited variants in LRS and SRS

For comparing substitutions and indels between LRS and SRS, we used bcftools isec (version 1.8) [42] to generate the intersection of two call sets. For the detection of Mendelian inheritance errors, we used vcftools (version 0.13) with –mendel option [43]. For comparing structural variant call sets between two platforms, we used the Truvari (version 3.5) “bench” command using default parameters [44].

### Comparison of small de novo mutations in LRS and SRS

For the comparison of the small DNMs, we performed two analyses. First, we compared LRS small DNMs with SRS small DNMs. DNMs on the LRS strict list were overlapped with small DNMs on the SRS list. These mutations were marked as “overlap.” Then, mutations were overlapped with the in-house unique and DeNovoCNN unique lists. Resulting variants were marked as “LRS + ,” while remaining mutations were marked as “LRS-unique.”

Subsequently, we compared the SRS variants with the LRS variants. DNMs on the SRS list were overlapped with the small DNMs on the strict LRS list. These variants were marked as “overlap.” Then, variants were overlapped with the lenient LRS list and resulting variants were marked “SRS + .” Finally, remaining variants were marked as “SRS-unique.”

### Clustered small de novo mutations

During LRS small DNM analysis, we identified clusters of non-overlapping variants that fall in the same gene with approximately the same coverage and variant allele frequency. Since these variants were all unique to LRS and appeared inherited when checking the read alignment in IGV, we decided to systematically remove these clustered DNMs. In order to do this, we first selected LRS-unique variants separated per trio and ordered by the chromosome and genomic position. Then, variants in resulting lists were marked if the gene name was the same as the gene name of the previous and/or next variant on the list. The same was done for intergenic variants. Subsequently, clusters of DNMs were defined when two or more variants fell within one average read length from each other (Additional file 1: Table S2A). Clustered DNMs were excluded from further analyses.

### Substitution and indel validation

All 54 LRS-unique, and 42 of the 133 SRS-unique variants were attempted to be validated using Sanger sequencing of proband, mother, and father. Primers were designed using Primer3Input. For 27 of the LRS-unique DNMs, we were not able to design a primer set, and these were not further validated. PCRs for the remaining 27 LRS-unique and 42 SRS-unique small DNMs were performed using Amplitaq Gold 360 Master Mix (Thermo Fisher Scientific) according to the manufacturer’s protocol. PCR products were enzymatically cleaned using Exonuclease I and FastAP, after which samples were Sanger sequenced. Finally, Sanger sequencing traces were analyzed using the SnapGene software package (version 5.2.2; GSL Biotech).

### STR validation

For 18 LRS-unique and 18 SRS-unique STR calls, we attempted validation using Sanger sequencing by the same approach as for the substitution and indel validations.

### Structural variant validation

All 23 LRS-unique SVs were validated using long-range PCR followed by sequencing on a PacBio Sequel IIe system. Primers were designed using Primer3Input, and PCR was performed using NEB LongAmp Hot Start Taq 2 × Master Mix. For each PCR product, 500 ng was used as input for the library preparation and the normalized library was prepared according to the manufacturer’s instructions using the SMRTbell barcoded adapter complete prep kit. Finally, the library, with a loading concentration of 80 pm, was sequenced on a PacBio Sequel IIe system using a Sequel II SMRT Cell 8 M (PacBio, Menlo Park, CA, USA) with a movie time of 30 h and 0.7 h pre-extension time.

### Titration analysis

For the comparison of our data to LRS data with lower coverage, downsampling was performed using SAMTools v1.10 [45]. Downsampling reduced the coverage of the samples from around 30 × to 20 × and 10 × . On these samples with reduced coverage, de novo calling was then repeated as described above. We then compared these de novo calls to a truth set consisting of variants validated by either SRS or Sanger sequencing.

### Phasing of small de novo mutations

For LRS, phasing was performed using WhatsHap [46], using the default options with the ‘–indels’ flag. Phased variants were considered informative for a de novo mutation if they are in the same phase block and were present in only one of the parents, while the other parent has homozygous reference call. Based on these informative variants, DNMs were classified as either paternal, maternal, or unknown, according to the following rules:

- If fewer than 3 informative variants were present on a haplotype of the candidate DNM, the DNM was considered unknown.
- If 3 or more informative variants were inherited from the same parent, then the DNM was assigned that respective parental origin. If more than 90% of the informative loci supported the same parental origin, the call was additionally classified as high quality.

For SRS sequencing data, we used GATK Haplotypecaller [47] to produce gVCFs. These were then combined with GATK CombineGVCFs tools and then genotyped with GATK GenotypeGVCFs. WhatsHap was run on the combined VCFs with default settings. DNMs were then classified according to the same rules as for LRS (described above).

## Results

**Fig. 1:**
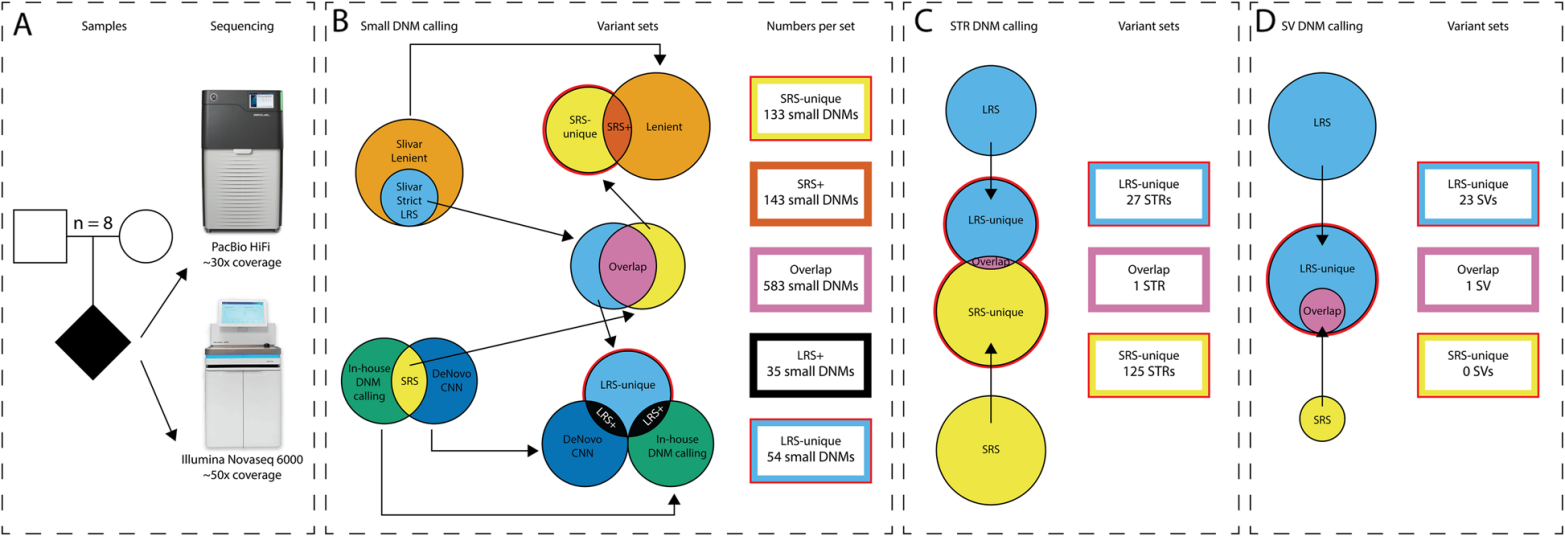
Study overview. We performed PacBio HiFi long-read sequencing and Illumina short-read sequencing for eight parent–child trios **A** De novo substitutions, indels, STRs, and SVs were called using dedicated bioinformatic tools. **B** For the small DNMs (substitutions and indels), the different call sets are depicted including the workflow of comparing the LRS call set to the SRS call set and the other way around. **C** The workflow for the comparison of STRs between LRS and SRS. **D** The workflow for the comparison of SVs between LRS and SRS. The circles in this figure are not drawn to scale

In order to demonstrate the utility of LRS for de novo mutation detection, we sequenced eight parent–child trios using high coverage PacBio HiFi sequencing. We previously performed Illumina short-read whole genome sequencing (SRS) for all eight trios [22], which allowed us to compare the performance of LRS and SRS on the detection of all types of variation in these trios, with a particular focus on de novo mutations (Fig. 1).

### Sequencing characteristics

For the PacBio HiFi LRS, we obtained average read lengths of 17 kb. Over 99.0% of the 5.7 million reads per sample aligned to the reference genome with an average mapping quality of 46.5 (Additional file 1: Table S2A). The base error rate, computed as the edit distance over total number of mapped bases, was 1.4% per sample (ranging from 1.2 to 1.5%), which is in agreement with what has been published before [48]. This resulted in an average coverage depth of 31 × for all 24 genomes, which was as expected based on targeted coverage of 30 × . 92.6% of the genome had at least 10 × coverage depth. The average read mapping rate for the SRS was 99.6% with an error rate of 0.9% (ranging from 0.8 to 1.0%; Additional file 1: Table S2B). The average coverage depth was 73 × (ranging from 51 × to 103 ×), and across all samples 83.4% of all bases were covered ≥ 50 × , while 92.0% were covered ≥ 10 × .

### Variants overview

Variant calling from LRS with DeepVariant and from SRS with GATK both yielded on average 4.1 million substitutions per sample (Additional file 1: Table S3A). On average, 3.8 million substitutions per sample were shared between two platforms, which corresponds to 94.0% concordance for both the LRS and SRS call sets (Additional file 1: Table S3A). Of the substitutions that are unique to LRS, about half of all LRS-unique variants (average 110,000), was detected in regions for which SRS had no read coverage (Additional file 1: Table S3A). We found that LRS provides sequence coverage in about 240 Mb of the genome where SRS does not. We found that in these regions the rate of Mendelian inheritance errors for LRS is only 2.1% suggesting that the majority of variant calls are real (Additional file 1: Table S3A).

For indels, the same callers yielded on average 1.0 million variants for LRS compared to an average of 0.9 million indels per sample with SRS (Additional file 1: Table S3B). The concordance was only 63.1% for SRS and 58.0% for the LRS indel call set (Additional file 1: Table S3B). For indels unique to LRS, around 25% were detected in regions that SRS had no read coverage (Additional file 1: Table S3B). The MIE ratio of LRS-unique indels (8.9%) was lower than that of SRS-unique indels (13.0%), indicating a slightly better ability of LRS for detecting indels (Additional file 1: Table S3B).

### De novo small variant detection

Performing both LRS and SRS on the same samples allowed us to identify all variant types including substitutions. In this study, we focused on assessing the accuracy of LRS HiFi for comprehensively calling small variants and SVs. A sensitive way of doing this is to detect and assess de novo mutations, since this type of variation has proven to be an important factor in the disease etiology of severe, early-onset, rare disease.

**Fig. 2:**
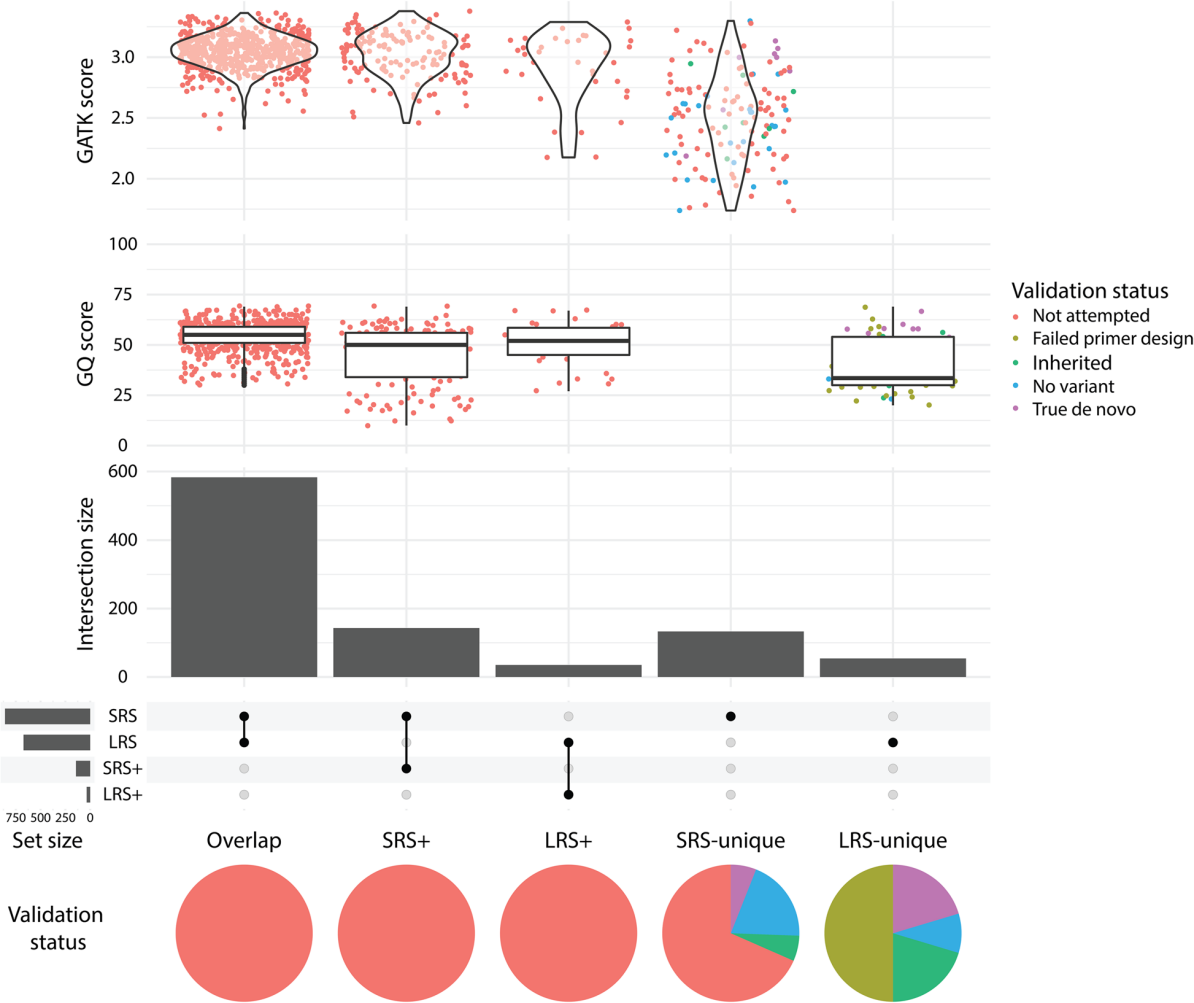
Detailed overview of all small DNMs. Upset plot of small DNMs detected by SRS and LRS. The *X*-axis shows the concordance across different call sets. The *Y*-axis shows, from bottom to top, number of DNMs in each group, the DeepVariant Genotype Quality (GQ) scores of these small DNMs from the LRS data and the log-scaled GATK quality scores of these DNMs from the SRS data. Colors indicate the validation status and pie charts show the validation status of DNMs in each group

During LRS small DNM analysis, we identified clusters of LRS-unique variants that fall in the same gene with approximately the same coverage and variant allele frequency. These variants all appeared not de novo upon visual inspection and were removed from further analyses as described in more detail in the “Methods.” In total, 672 small DNMs were identified using strict filtering criteria, with on average 84 (range 73–92) small de novo mutations per child using PacBio HiFi LRS (Fig. 1B; Additional file 1: Table S4A and Additional file 2: Table S5), being in line with previously reported number of de novo substitutions per genome [2, 3]. On average, 75 of these 84 variants were single base substitutions, while there were 4 insertions and 5 deletions between 2 and 50 bp. Only two insertions > 50 bp were called using DeepVariant and these were also retained. Comparison of small DNMs called from LRS data versus substitutions called from SRS data showed 92.0% concordance (Fig. 2; Additional file 1: Table S6A). Of the overlapping substitutions, 94.3% were called by both SRS DNM callers in the overlap set and the other 5.7% by only one of the SRS DNM callers in the LRS + set (see “Methods”). Among all LRS DNM call sets, eleven were located in the coding regions of the genome and were all detected by both LRS and SRS (Additional file 3: Table S7). For SRS, we found 859 small DNMs, with on average 107 (range 91–141) small DNMs per patient (Fig. 1B; Additional file 1: Table S4B and Additional file 4: Table S8), including 95 substitutions, 4 insertions, and 8 deletions. The concordance for SRS small de novo mutations versus those called from LRS data was 84.5% (Additional file 1: Table S6B). Of the overlapping small DNMs, 80.3% were called using the stringent LRS de novo filtering in the direct overlap set and the other 19.7% using the lenient LRS de novo filtering in the SRS + set (see “Methods”). The concordance for coding small DNMs was 100% (13/13 variants; Additional file 3: Table S7), albeit that 2 of the 13 coding variants were only identified in LRS after lenient filtering.

### Small de novo mutation validation

In order to assess the sensitivity of LRS for the detection of small DNMs, we first attempted to validate all 54 LRS-unique small de novo mutation (Additional file 2: Table S5 and Additional file 1: S9A; Additional file 1: Fig. S1). For these 54, we aimed to design standard primer pairs suitable for Sanger sequencing. Due to the complex genomic regions of these variants, we only succeeded to design primers for 27 (50%) of the 54 variants. Of the 27 variants with successful primer design, 11 (40.7%) were confirmed as a true DNM, 11 were true variants but inherited from one of the parents, and five were not confirmed in the child and therefore considered false positives (Fig. 2; Additional file 2: Table S5). Small DNM quality scores were on average significantly higher for the confirmed DNM calls than for the inherited and false positive calls with quality scores of 55.5 and 54.4 for confirmed vs. 36.6 and 36.5 for inherited variants vs. 31.2 and 30.6 for false positive variants (*P* = 2.8e − 7, *P* = 8.3e − 6, *P* = 8.3e − 6, and *P* = 3.0e − 4; *t*-test) (Additional file 1: Fig. S2). When looking at the specific locations of the 11 confirmed LRS-unique small DNMs in the SRS data, we found that all DNMs showed coverage at the specific genomic position and that the mutations were called. However, ten of these mutations were assessed by the SRS de novo mutation callers as being potentially inherited due to a small number of alternative base calls in one of the parents, and one was assessed as low-quality DNM because of a small number of alternative base calls in one of the parents (Additional file 1: Table S10).

For the 133 SRS-unique DNMs, visual inspection of the reads at the specific genomic position identified seven of them as high-confidence candidate small DNMs. Others were identified as low-confidence candidate small DNMs, either due to low read support or repetitive reference context. For validation, we selected a set of 42 SRS-unique small DNMs including all seven high-confidence candidate small DNMs as well as 35 randomly selected low-confidence candidate small DNMs (Additional file 1: Table S9B). For the total of 42 small DNMs, we designed primers to determine whether these are true de novo calls. Of the 42 variants, eight (19%) were confirmed as true de novo events and eight other variants (19%) appeared to be inherited from one of the parents (Additional file 1: Fig. S3; Additional file 4: Table S8). For the remaining 26 variants (62%), Sanger sequencing failed to confirm the event called by Illumina SRS and these were therefore considered false positive calls. Five of the seven high-quality DNM candidates we initially selected were confirmed as true positive, while one had primer design sequencing failed and other one was false positive. Four of the eight SRS-unique true positive small DNMs also appeared de novo when inspecting the LRS alignment files (Additional file 1: Table S11). Of the four remaining SRS-unique small DNMs, two had low alternate allele depth and two had insufficient coverage for both alleles.

### Differences between substitutions and indels

The predominant error mode in long-read sequencing is short insertions and deletions [10]. We therefore investigated whether there was a difference for the detection of substitutions and indels for both platforms. The 27 LRS-unique small DNMs for which we were able to perform validations consisted of 13 substitutions, 10 insertions, and 4 deletions. None of the insertions and deletions were confirmed as true DNMs, while 11 substitutions were confirmed true de novo. For the insertions and deletions, 70 and 75% were inherited from one of the parents while 30 and 25% were false positive variant calls respectively. In addition, the average quality scores for the substitutions, insertions, and deletions were divergent (53.5 and 52.5 vs. 32.9 and 32.5 vs. 36.3 and 36.5) (Additional file 1: Fig. S1). For the SRS-unique variants, the 42 validated variants consisted of 34 substitutions, 2 insertions, and 6 deletions. Only one deletion and seven substitutions were confirmed as true DNMs. Both insertions were false positive calls. In general, the SRS-unique variants were enriched for false positive calls, since 68% of the substitutions, 100% of the insertions, and 17% of the deletions were false positive variant calls (Additional file 1: Fig. S3). Furthermore, 9.5% of the substitutions were inherited while this was 0% for the insertions and 83% for the deletions.

### De novo STRs

For STRs, we used a tandem repeat catalog, containing 171,146 highly polymorphic repeat loci, as input for both TRGT and ExpansionHunter for LRS and SRS, respectively. On average, we genotyped both alleles of all three family members for 171,038 (99.93%) loci for LRS and 171,113 (99.98%) for SRS (Additional file 1: Table S12). In total, we identified 28 (mean 4; range 1–6; Fig. 1C and Additional file 5: Table S13) and 126 (mean 16; range 5–31; Fig. 1C and Additional file 6: Table S14) repeat loci in LRS and SRS where one or both alleles in the child were ≥ 2 repeat units longer or shorter than the number of repeat units in both parents and met our quality metrics (“Methods”). Therefore, these repeat calls were considered high-quality candidate de novo STRs. Of these de novo repeats, only one call (3.6% for LRS and 0.8% for SRS) was concordant between the two platforms (Additional file 1: Table S15). We attempted to validate 18 LRS-unique and 18 SRS-unique high-quality de novo STR calls. For the LRS-unique calls, none were confirmed as true de novo repeat expansion. Of the 18 STR calls, 14 were false positive calls and four were true but not de novo because the repeat length was the same in one or both parents (Additional file 1: Table S16). For the SRS-unique STRs also, none of the 18 high-quality de novo STR calls were confirmed as true de novo as 13 calls were false positive and five were true but inherited from one or both of the parents (Additional file 1: Table S16).

### De novo SVs

**Fig. 3:**
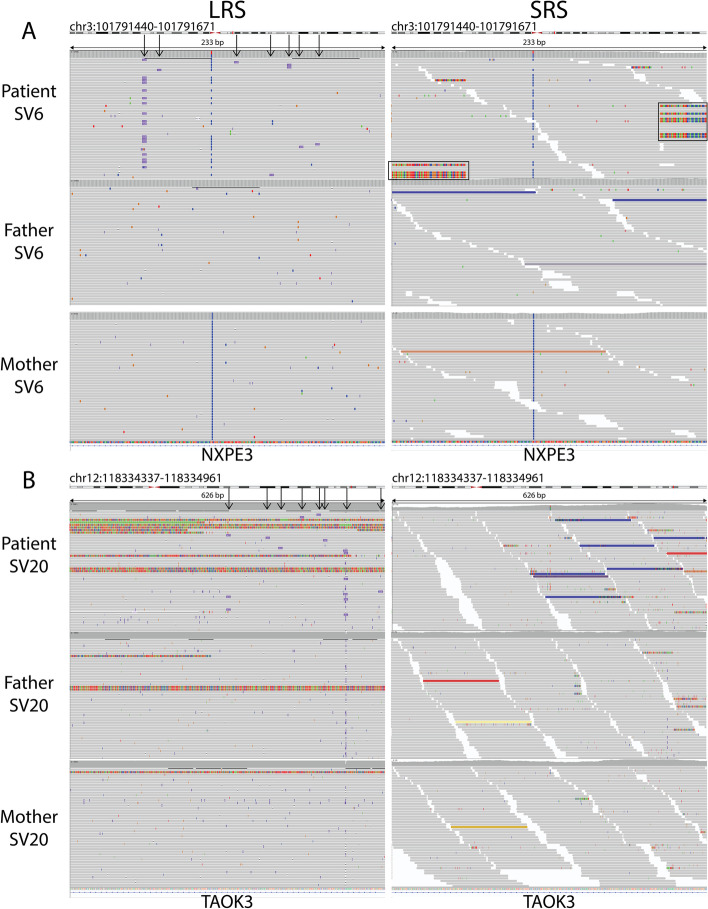
Two confirmed true de novo SVs only detect by LRS. For two variants, the gel image and Integrative Genomics Viewer (IGV) screenshot for LRS and SRS is presented. **A** SV6 is a 123 bp duplication in an intron of the gene *NXPE3*. The position of the duplication call in the child’s LRS reads is indicated with a black arrow. In the SRS data, there are clipped reads, indicated with the two red boxes, hinting towards an SV event. However, the SV was not called in SRS. **B** SV17 is a 303-bp insertion in an intron of the gene *TAOK3*. The position of the insertion call in the child’s LRS reads is indicated with a black arrow. In the SRS data, some blue reads are visible, which represent reads with a smaller insert size than expected indicating a possible insertion. However, the SV was not called from the SRS data

In addition to substitutions, indels, and STRs, we also identified de novo structural variants for our patients using PBSV for LRS and Manta for SRS. In total, we identified 24 de novo candidate SVs with LRS and one de novo candidate SV with SRS (Fig. 1D; Additional file 7: Table S17). The one SV in SRS that overlapped with LRS and was considered a true de novo event. The remaining 23 LRS-unique variants consisted of 13 insertions, 8 deletions, and 2 duplications (size range 21–991 bp). We aimed to systematically validate the de novo SVs using (long-range) PCR and subsequent targeted sequencing on a PacBio Sequel IIe system. For four of 23 variants, validation experiments repeatedly failed due to difficulties with designing suitable PCR primers. However, two out of the 23 variants were confirmed as genuine de novo SV events (Fig. 3). In addition, eight SVs (five insertions and three deletions) in the size range of 21 to 991 bp were located within repeat regions and could be considered as de novo repeat expansions and contractions (Additional file 1: Fig. S4). Therefore, in total 10 SVs were confirmed as a de novo event. Out of the 9 events that were not de novo, two SVs were inherited from one or both parents, while seven events were not detected at all (Additional file 7: Table S17). When analyzing the alignment files of both LRS and SRS at the genomic positions of all 24 SVs, it turned out that, besides the one overlapping SV, seven different LRS-unique SVs could be visually detected in hindsight (Additional file 7: Table S17; Additional file: Fig. S5). Of these, five were validated as de novo event and one was inherited, while for one multiple validation attempts failed. For the remaining 16 SVs, we did not observe any patterns reminiscent of an SV in the SRS data (Additional file 7: Table S17; Additional file 1: Fig. S5).

### Titration

Our LRS samples were sequenced to relatively high coverage depth of 30 × . When downsampling to an average 20 × and 10 × coverage depth for the child and parents, we observed on average 8 and 37 out of 75 validated DNMs could no longer be detected in the proband respectively (Additional file 1: Table S18; Additional file 1: Fig. S6). In addition, the number of potential small DNMs increased considerably to on average 234 and 1120 calls at 20 × and 10 × coverage depth respectively. This suggests that with the current LRS technology obtaining 30 × average coverage depth is required for optimal detection of DNMs.

### Phasing of small de novo mutations

**Fig. 4:**
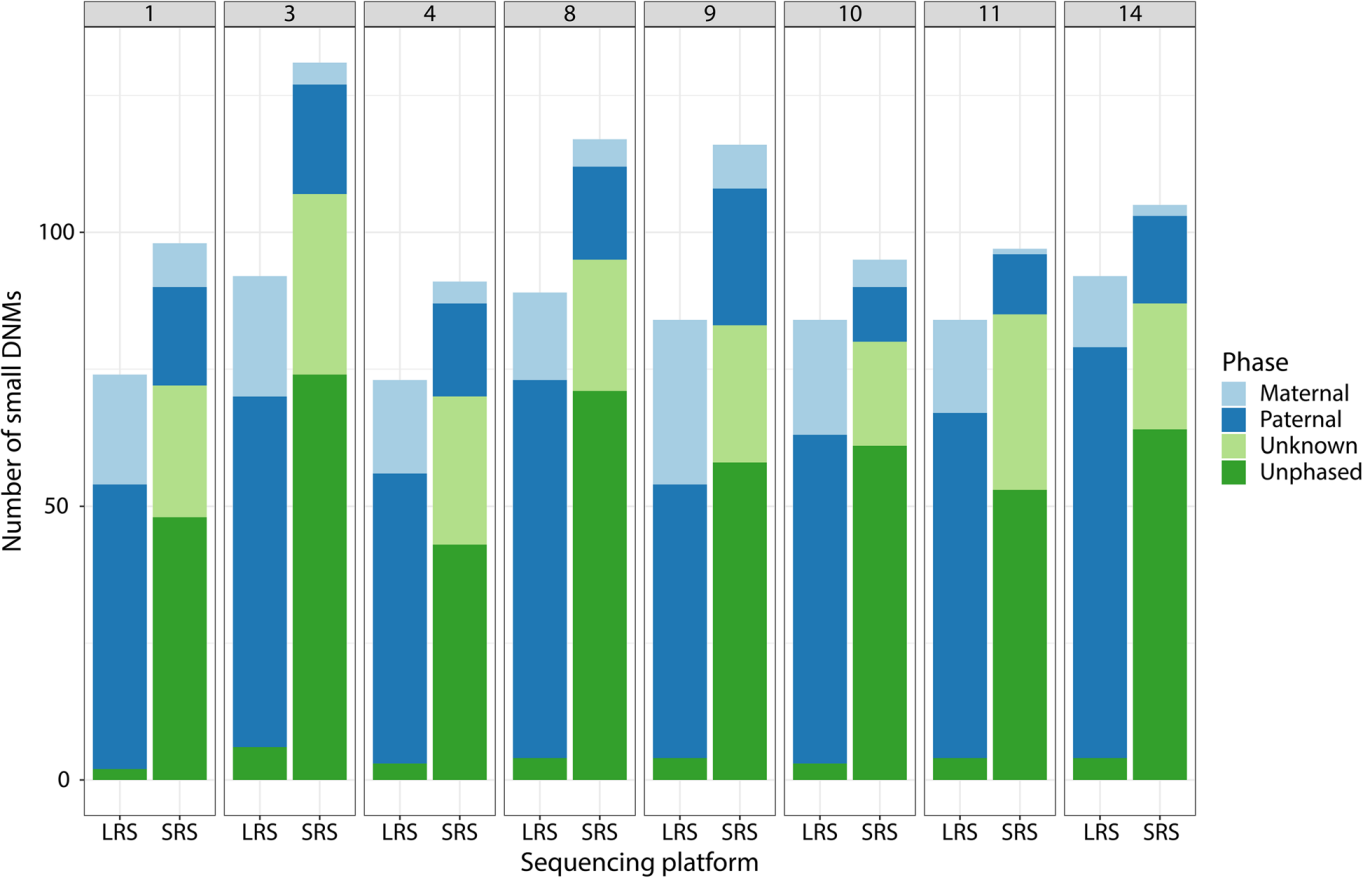
Phasing of DNMs. Number of DNMs per trio in a stacked bar graph, with colors for phasing results. With SRS and LRS next to each other grouped per trio (on average 89 phased small DNMs in LRS versus 21 phased small DNMs in SRS). Status of parentally phased DNMs for each trio. *X*-axis shows the sequencing platform, while *Y*-axis shows the number of DNMs. Colors indicate the assigned parental origin

One potential advantage for the detection of small DNMs using long reads is the possibility to phase the DNMs and determine the parent-of-origin based on inherited variants (i.e., markers; Fig. 4). Using the LRS reads for phasing resulted in haplotype blocks with a mean length of 570 kb, with an average of 800 small variants per block. When we used the SRS reads, mean length of haplotype blocks was 1.2 kB, with 14 small variants per block. With LRS, we were able to assign 96% of DNMs to a haplotype block and subsequently all DNMs with a haplotype block were assigned to a parental allele (Additional file 1: Table S19A). For more than 80% of the phased DNMs from LRS, there was > 90% agreement between markers. With SRS, we were able to assign 46% of the DNMs to a haplotype block. Because of the relatively small size of the phase blocks, we could only assign 20% of the total DNMs to a parental allele (Additional file 1: Table S19B). Comparing the successfully phased DNMs, we found > 90% concordance between SRS and LRS (Additional file 8: Table S20). We found that all three discordant DNMs (100%) were found in repeated and low complexity regions of the genome. We found that 72.3% of the phased small LRS-detected DNMs and 78.4% of small SRS-detected DNMs were paternal, which was expected based on other studies of DNMs [5, 49, 50] (Additional file 1: Table S19A).

One of the advantages of performing phasing is that we expect that true DNMs to be phased with high quality, while false positives caused by sequencing artifacts would not fit into a haplotype. Therefore, we checked the phasing of true positive and false positive DNMs from the LRS call set. All 11 validated LRS-unique small DNMs were phased. For the false positive and inherited DNM calls, 13 of 16 were not successfully assigned to a haplotype block by Whatshap (Additional file 1: Table S21). These results show that phasing can help to distinguish true positive from false positive DNMs (Fisher’s exact test, *P* = 3.39e − 5).

## Discussion

Here we investigated whether HiFi PacBio sequencing offers sufficient base call quality to allow for comprehensive de novo mutation detection. This is important for several reasons: LRS is currently considered for samples that were unresolved by exome or genome sequencing with the intention to identify so far “hidden,” undetected, SVs. However, several studies have shown that short-read exomes or genomes may have missed genetic variants due to limitations of the technology or experimental design at that time [10, 13, 14, 51]. The potential ability to comprehensively detect all types of variation, now enabling the technically challenging de novo mutations, paves the way for LRS to replace SRS as the standard technology for genetic analyses as soon as costs become comparable. This possibly enables testing of “all” rare disease patients with a suspected genetic cause with a single comprehensive test. However, depending on platform and local infrastructure, LRS is currently 3–sixfold more expensive than SRS.

When comparing all substitutions between the platforms, we find that there is a 94% concordance and that the MIE rate for variants unique to LRS is very low at 2.1%. If we assume that all substitutions with correct Mendelian inheritance are real, the average sensitivity of HiFi LRS is 99.84% compared to 99.96% for SRS, and for indels 95.03 and 93.44% respectively. When comparing DNMs from LRS with SRS, we find that the overlap for LRS was 92.0% and for SRS 84.5%. LRS detected 54 unique variants while SRS detected 133 unique variants. With SRS, we found that these specific variants are mostly false positive calls in the proband (i.e., sequencing and mapping artifacts). Our false positive rate is higher than what should be expected for routine genetic testing applications where typically additional quality filtering will be applied. For LRS, most variants (68.8%) were true but were inherited from one of the parents. The same observation was made by Noyes et al. in their study that used multiple long- and short-read technologies to establish a most comprehensive set of DNMs in a parent–child quad [5]. They observed that 71% of false DNM calls were due to a missed call in one of the parents. This is promising for LRS technology since improved coverage, or improved evenness of coverage, will likely reduce this type of false positives. If we would disregard this type of false positives from our study, LRS performs very favorably to SRS. In this case, our de novo validation rate for LRS, considering only successful PCRs, would increase from 40.7% (11/27) to 68.8% (11/16), while the validation rate for SRS DNMs would only increase from 19.0% (8/42) to 23.5% (8/34).

We experimentally validated 11 LRS-unique and 8 SRS-unique DNMs. For the LRS-unique variants, we found that all 11 were called by SRS as well. One DNM was assessed as low-quality DNM in SRS due to mapping quality issues at the position of this event. The other ten were considered as potentially inherited mutations since they had fractional support (3–6 reads with alternative allele) in the parents. This might be due to sequencing artifacts or might be due to parental mosaicism. For two of these ten variants, Sanger sequencing traces only showed a small variant peak in one of the parents. Therefore, we still considered these as true DNMs, but the deeper sequence coverage of SRS had a slightly higher chance to identify low parental mosaicism in these two. For the SRS-unique small DNMs, four out of the eight were not called de novo in LRS due to poor genotyping in one of the parents. Two small DNMs were not called de novo in LRS due to the presence of alternative allele reads in one of the parents. The remaining two small DNMs were not called in LRS due to insufficient coverage and a low variant allele frequency at the position of the event in the proband.

Compared to the study by Noyes et al., our per-sample de novo mutation numbers and concordance ratio (between SRS and LRS) are very similar [5]. Noyes et al. called an average of 81 de novo substitutions and 6 indels in their probands. Our per-sample averages for these types of variants were 75 and 9 respectively. On the other hand, their SRS call set consists of on average 82 de novo mutations in the probands, which is somewhat smaller than our per-sample average of 107. This is mostly because de novo mutation*s* in repetitive regions were removed from their call set. Since their SRS call set is more restrictive, the concordance of their LRS call set compared to their SRS call set drops under 80%, compared to our finding of 92.0%.

In a previous study, we applied LRS to 5 trios, using a PacBio Sequel system and without the use of circular consensus calling to improve base pair accuracy [4]. When considering the single trio from this study that was sequenced at similar coverage (30 ×), we previously identified 655 small DNM candidates compared to 84 small DNMs per trio in our current study. Even though, due to the higher error rate in the sequencing data of our previous study [4], the selection criteria for small DNMs were much more stringent, the number of small DNM candidates was still considerably higher. In our comparison with SRS, we identified an overlap of only 58.3% whereas in our current study this is 92.0%. Fifty percent of false positive small DNMs in our previous study were due to false positive insertion calls in the proband, whereas this is a substantially smaller proportion (30%) in the current study. The differences between these studies illustrate the improvements that have been made with the introduction of HiFi sequencing.

Besides showing that small DNM calling using HiFi LRS is *on par* with SRS, we have also analyzed four traditional benefits of LRS over SRS. First, we show the more accurate detection of de novo SVs. We confirmed ten out of 23 de novo SVs in total, of which eight in Repbase [52] annotated repetitive DNA elements. The validation rate of only 43.5% may be explained in part due to the proximity of all 23 de novo SVs to repetitive reference contexts which made these challenging to confirm. Out of the 13 SVs that were not confirmed, for four the validation did not refute the de novo event itself but was mostly inconclusive. Combining the ten confirmed de novo SVs with the one SV detected by both sequencing platforms this comes down to an average of 1.375 de novo SV per genome. This number is markedly higher than current estimates based on short-read WGS data of 0.02 to 0.286 de novo CNVs and SVs (> 50 bp) per genome [53–55]. This is likely due to detection of the eight SVs that could be considered repeat expansions/contractions. When only considering the other three SVs, our study also suggests that de novo SVs are rare with a de novo SV rate of 0.375. The rare nature and the possibility to identify those reliably with LRS, without enriching for false positive SV calls, confirms the accuracy of LRS for this variant class. It also confirms that a de novo SV hypothesis for rare and severe disease works and strongly reduces the number of candidate variants per offspring.

Secondly, we performed STR detection from both LRS and SRS. In total, we detected 28 high-quality de novo STR expansions and contractions for LRS and 126 for SRS. Of these, only one overlapped between the two technologies. Moreover, none of the LRS-unique and SRS-unique repeat expansion, for which we attempted validations, was confirmed to be true de novo. This shows that although LRS seems to outperform SRS in this area with a slightly higher specificity, there is still a lot of room for improvement in the detection of STRs. For a fair comparison, we restricted our analysis to 171,146 known highly polymorphic repeat regions. However, to show the full potential of the detection of STRs with LRS, a genome-wide analysis would be more appropriate. This is illustrated by the fact that the SV calling on LRS data identified another eight de novo structural variants that upon closer examination turned out to be STR expansions/contractions.

Thirdly, LRS allows improved phasing of the DNMs and determine the parent-of-origin based on surrounding inherited variants. Comparing only phased small DNMs, we find a more than 90% concordance of the assigned parental allele between LRS and SRS. However, almost all DNMs (> 96%) could be phased with LRS compared to only 20% with SRS. Phasing also supports the quality of our DNM results in LRS. The fact that the DNMs are not artifacts is supported by the consistency of the phasing by multiple single-nucleotide polymorphisms, all supporting the same parental allele. In LRS, additional validation of a DNM with an orthogonal technology could be omitted when additional support from phasing results based on a reasonable number of SNPs is available. Benefits of phasing in future studies not only entail this increase in DNM specificity, but could also increase the specificity for post-zygotic and somatic DNMs [7, 56] and allow better studies of DNM biology [49, 57].

Finally, with LRS more of the human genome is accessible and, for the first time, variants can be called in these regions that remained inaccessible with other sequencing technologies. With LRS we found on average 240 Mb of uniquely covered regions per sample, compared to 133 Mb per sample for SRS. This is also in agreement with previous literature about the dark regions of the genome [58].

Despite these advantages of LRS over SRS, the cost of sequencing is an important disadvantage of HiFi LRS. The current price of a HiFi genome at 30-fold coverage is 3–6 times higher than a genome achievable with SRS at 30-fold coverage. With future iterations of the HiFi LRS platform the costs for a 30-fold coverage genome will drop up to threefold, but also SRS will be available at half its current price. To address whether the benefits of LRS are worth the additional costs, more extensive clinical utility studies are required, which is beyond the scope of this current study.

## Conclusions

HiFi LRS can now produce a very comprehensive WGS dataset obtainable by a single technology in a single laboratory, allowing accurate calling of substitutions, indels, STRs and SVs. This possibly enables for truly generic testing of “all” rare disease patients with a suspected genetic cause with a single comprehensive test. The accuracy of HiFi LRS even allows sensitive calling and phasing of DNMs, which are a major cause of severe early-onset disease, on all variant levels.

## Supplementary Information

**Additional file 1: Fig. S1.** Overview of validated LRS-unique small de novo mutation calls. **Fig. S2.** LRS quality scores for validated LRS-unique small DNMs. **Fig. S3.** Overview of validated SRS small de novo mutation calls. **Fig. S4.** likely de novo repeat expansions and contractions. **Fig. S5.** A: Overview of the alignments of SV1-SV12 detected by LRS and SRS. For each SV this figure shows the SRS alignment in the top part and LRS alignment in the bottom part. Black arrows and boxes indicate where the SV is visible in the SRS and/or LRS alignments. B: Overview of the alignments of SV13-SV24 detected by LRS and SRS. For each SV this figure shows the SRS alignment in the top part and LRS alignment in the bottom part. Black arrows and boxes indicate where the SV is visible in the SRS and/or LRS alignments. **Fig. S6.** Titration results. **Table S1.** Overview of sequenced trios. **Table S2.** A. LRS statistics. B: SRS statistics. **Table S3.** A. SNV Calling overview. B: Indel variant calling overview. **Table S4.** A: Summary of the number of identified de novo substitution in LRS. B: Summary of the number of identified de novo substitution in SRS. **Table S6.** A: Overlap between LRS and SRS small DNMs. B: Overlap between SRS and LRS small DNMs. **Table S9.** A: Validation overview LRS-unique small DNMs. B: Validation overview SRS-unique small DNMs. **Table S10.** LRS-unique small DNMs. **Table S11.** SRS-unique small DNMs. **Table S12.** A: STRs in LRS data. B: STRs in SRS data. **Table S15.** Overlap between LRS and SRS for de novo STRs. **Table S16.** Validation overview for LRS-unique and SRS-unique de novo STRs. **Table S18.** Effect of coverage on DNM detection. **Table S19.** A: Phasing of LRS detected DNMs and parental age. B: Phasing of SRS detected DNMs. **Table S21.** Phasing details of small DNMs uniquely detected by LRS.

**Additional file 2: Table S5.** All small DNMs detected by LRS.

**Additional file 3: Table S7.** Coding small de novo mutations detected by LRS and SRS.

**Additional file 4: Table S8.** All small DNMs detected by SRS.

**Additional file 5: Table S13.** Overview of all high quality de novo STRs in LRS.

**Additional file 6: Table S14.** Overview of all high quality de novo STRs in SRS.

**Additional file 7: Table S17.** Overview of SVs in LRS and SRS.

**Additional file 8: Table S20.** Phasing details of small DNMs detected by both LRS AQand SRS.

## Data Availability

The datasets obtained from individuals with biobank consent include approval for data sharing in EGA, including six complete trios and one singleton proband, uploaded under accession number EGAS00001006479 [59]. Re-use of the data will be evaluated by a data access committee to evaluate whether the proposed re-use is in line with consent obtained. For the one remaining proband and four unaffected parents, consent did not allow upload to EGA, and similarly broad re-use of data is limited. Nonetheless, all data obtained of relevance to support the conclusions are presented in the supplementary datafiles, for which more details are available upon reasonable request from the authors.

## Abbreviations

AQ: Allele quality
BAM: Binary Alignment Map
CCS: Circular consensus sequencing
CNV: Copy number variant
DNM: De novo mutation
GATK: Genome Analysis Toolkit
GQ: Genotype quality
HiFi: High fidelity
IGV: Integrative Genome Viewer
Indel: Insertion or deletion
LRS: Long-read sequencing
PBSV: Pacific Biosciences Structural Variant
PCR: Polymerase chain reaction
SMRT: Single-molecule real-time
SRS: Short-read sequencing
SV: Structural variant
TRGT: Tandem Repeat GenoTyper
WGS: Whole genome sequencing
