# Supplementary material for: Comprehensive de novo mutation discovery with HiFi long-read sequencing
Source: Genome Med. 2023 May 8;15:34. doi: 10.1186/s13073-023-01183-6 (PMC10169305; doi:10.1186/s13073-023-01183-6)
Supplement: Supplementary file 1 — Additional file 1: Fig. S1. Overview of validated LRS-unique small de novo mutation calls. Fig. S2. LRS quality scores for validated LRS-unique small DNMs. Fig. S3. Overview of validated SRS small de novo mutation calls. Fig. S4. likely de novo repeat expansions and contractions. Fig. S5. A: Overview of the alignments of SV1-SV12 detected by LRS and SRS. For each SV this figure shows the SRS alignment in the top part and LRS alignment in the bottom part. Black arrows and boxes indicate where the SV is visible in the SRS and/or LRS alignments. B: Overview of the alignments of SV13-SV24 detected by LRS and SRS. For each SV this figure shows the SRS alignment in the top part and LRS alignment in the bottom part. Black arrows and boxes indicate where the SV is visible in the SRS and/or LRS alignments. Fig. S6. Titration results. Table S1. Overview of sequenced trios. Table S2. A. LRS statistics. B: SRS statistics. Table S3. A. SNV Calling overview. B: Indel variant calling overview. Table S4. A: Summary of the number of identified de novo substitution in LRS. B: Summary of the number of identified de novo substitution in SRS. Table S6. A: Overlap between LRS and SRS small DNMs. B: Overlap between SRS and LRS small DNMs. Table S9. A: Validation overview LRS-unique small DNMs. B: Validation overview SRS-unique small DNMs. Table S10. LRS-unique small DNMs. Table S11. SRS-unique small DNMs. Table S12. A: STRs in LRS data. B: STRs in SRS data. Table S15. Overlap between LRS and SRS for de novo STRs. Table S16. Validation overview for LRS-unique and SRS-unique de novo STRs. Table S18. Effect of coverage on DNM detection. Table S19. A: Phasing of LRS detected DNMs and parental age. B: Phasing of SRS detected DNMs. Table S21. Phasing details of small DNMs uniquely detected by LRS. [file 13073_2023_1183_MOESM1_ESM.pdf]

Additional file 1

Supplement to:

Comprehensive *de novo* mutation discovery with HiFi long-read sequencing

Erdi Kucuk[1,2*], Bart P.G.H. van der Sanden[1,3*], Luke O'Gorman[1], Michael Kwint[1], Ronny Derks[1], Aaron M. Wenger[4], Christine Lambert[4], Shreyasee Chakraborty[4], Primo Baybayan[4], William J. Rowell[4], Han G. Brunner[1,3,5,6], Lisenka E.L.M. Vissers[1,3], Alexander Hoischen[1,2,7,*], Christian Gilissen[1,2,*]

[1]Department of Human Genetics, Radboud University Medical Center, Nijmegen, The Netherlands
[2]Radboud Institute for Molecular Life Sciences, Radboud University Medical Center, Nijmegen, The Netherlands
[3]Donders Institute for Brain, Cognition and Behaviour, Radboud University Medical Center, Nijmegen, The Netherlands
[4]Pacific Biosciences, Menlo Park, CA, USA
[5]Department of Clinical Genetics, Maastricht University Medical Center, Maastricht, The Netherlands
[6]GROW School for Oncology and Developmental Biology, Maastricht University Medical Center, Maastricht, The Netherlands
[7]Radboud University Medical Center for Infectious Diseases (RCI), Department of Internal Medicine, Radboud University Medical Center, Nijmegen, the Netherlands

[*]These authors contributed equally

Corresponding authors:

Christian Gilissen, PhD
Email: christian.gilissen@radboudumc.nl

Alexander Hoischen, PhD
Email: alexander.hoischen@radboudumc.nl

Radboudumc, Department of Human Genetics
PO Box 9101, 6500 HB
Nijmegen, the Netherlands

Best practices

In order to get the best results for the comprehensive detection of *de novo* mutations using PacBio HiFi long-read sequencing we advise to:

- Apply coverage filters to increase accuracy for substitutions and small indels
- Remove clustered substitutions and small indels to reduce the number of false positive calls
- Phase mutations to get insight in the quality of the variant call
- Perform a visual inspection to assess small DNM quality and prioritize validations
- Apply quality filters for the detection of STRs

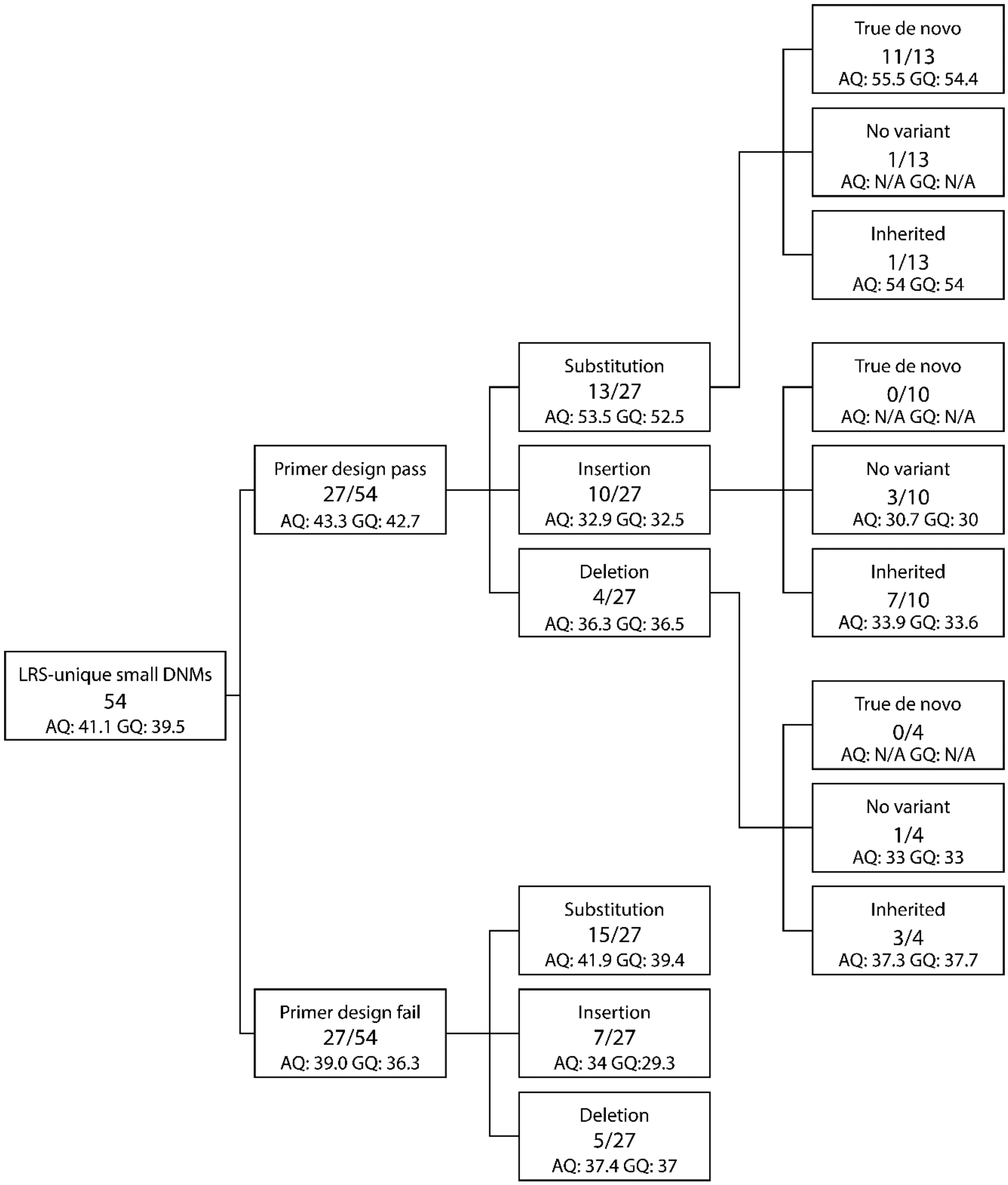

Fig. S1: **Overview of validated LRS-unique small *de novo* mutation calls** This figure shows the distribution of substitutions, insertions and deletions in the successful and unsuccessful validation group. For the small DNMs with successful Sanger validation also the validation status is presented per variant type. In addition, for all groups the allele quality (AQ) reflecting the quality of the alternate allele and genotype quality (GQ) reflecting the quality of the genotype is shown.

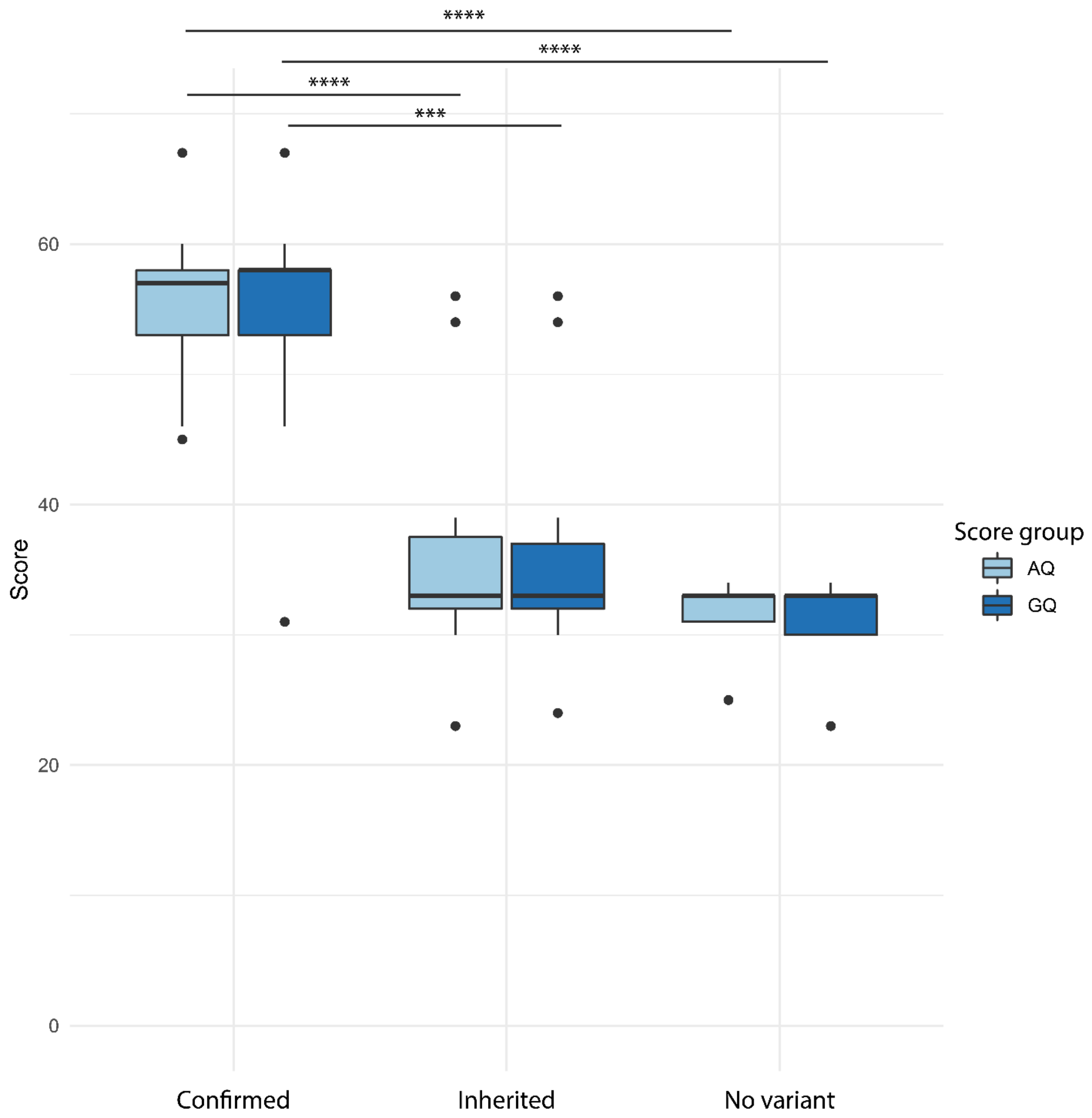

Fig. S2: **LRS quality scores for validated LRS-unique small DNMs**

For each validation category (x-axis) this plot shows the LRS allele quality score (AQ) reflecting the quality of the alternate allele and genotype quality (GQ) reflecting the quality of the genotype on the y-axis. AQ is shown in light blue and GQ is shown in dark blue. Means of the groups were compared using a t-test. P-values are P=2.8e-7, P=8.2e-6, P=8.2e-6 and P=3.0e-4. **** means P < 0.0001 and *** means P < 0.001.

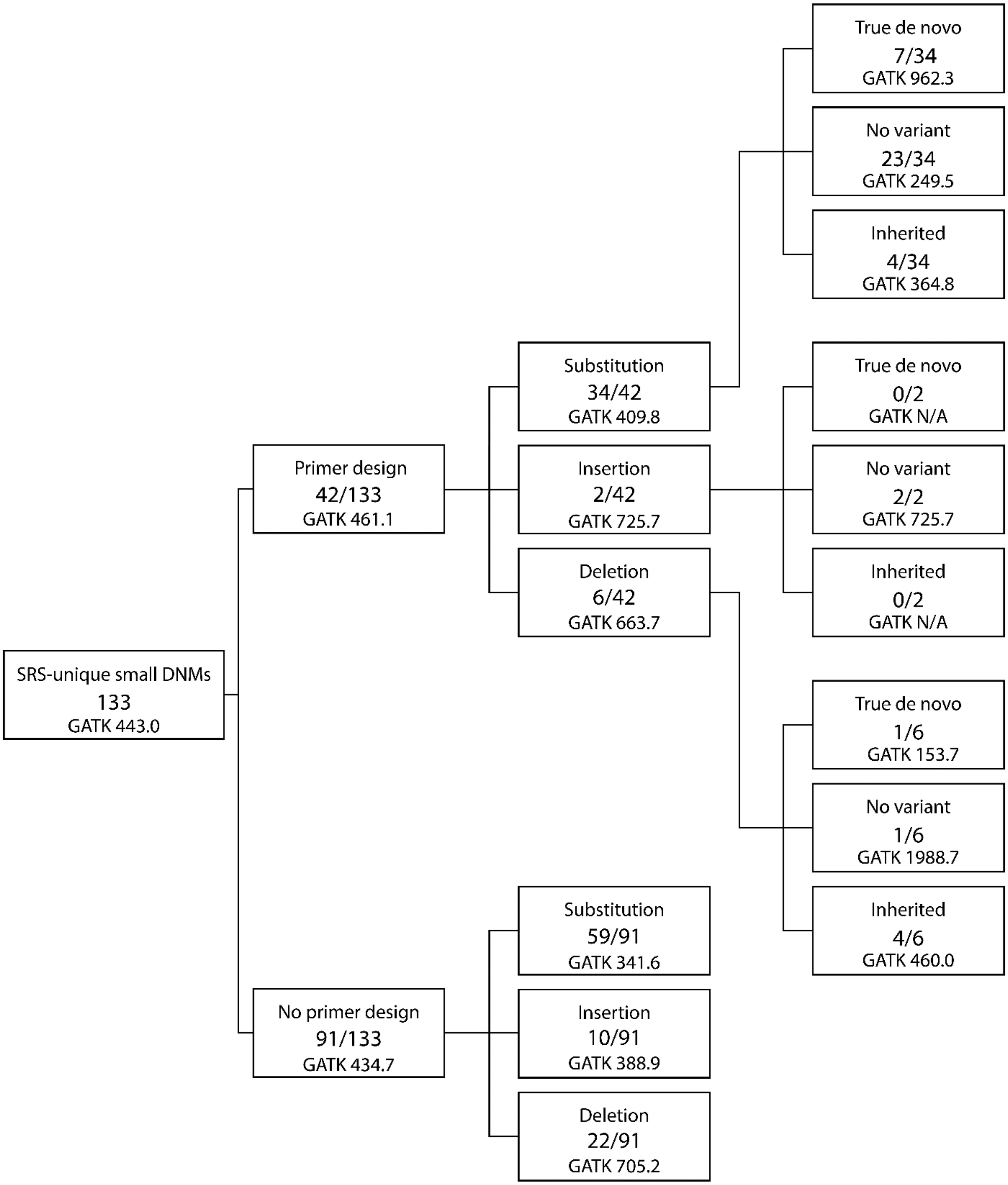

Fig. S3: Overview of validated SRS small *de novo* mutation calls

This figure shows the distribution of substitutions, insertions and deletions in the set of DNMs we selected for Sanger validations and the set that was not validated. For the validated DNMs also the validation status is presented per variant type. In addition, for all groups the GATK score is given. For the SRS-unique mutations we also performed visual inspection of the alignment files and only seven of the 133 mutations were considered a high quality DNM.

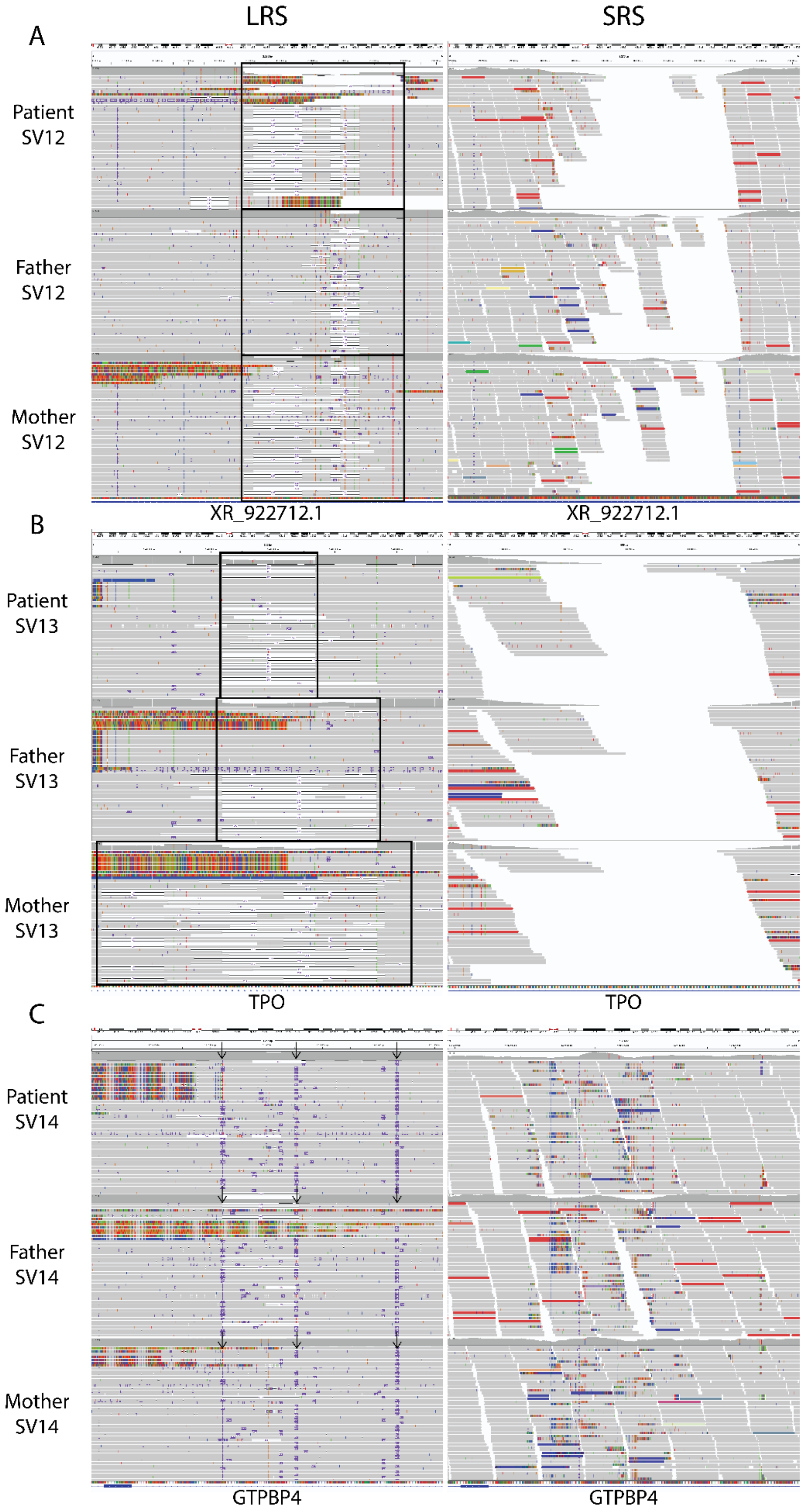
A
B
C
LRS
SRS
Patient SV12
Father SV12
Mother SV12
XR_922712.1
XR_922712.1
Patient SV13
Father SV13
Mother SV13
TPO
TPO
Patient SV14
Father SV14
Mother SV14
GTPBP4
GTPBP4

Fig. S4: likely de novo repeat expansions and contractions.

Three examples of the eight LRS-unique SVs where one or both of the parents had the same variant but with a different length. (A) SV12 is a 991 bp deletion in *XR_922712.1*. Also both parents have a deletion at this position, but the size is different. The deletion in LRS is depicted in the boxes. (B) SV13 is a 150 bp deletion in *TPO*. This SV is smaller than the deletion at the same position in both parents. The deletion is again depicted in the boxes. (C) SV14 is a 509 bp insertion in *GTPBP4*. The position of the insertion call in all family members' LRS reads are indicated with a black arrow. For these three SVs the SRS IGV image also indicate an event happening because of the red and blue colored reads. Red reads represent reads with a larger insert size than expected indicating a possible deletion. Blue reads represent reads with a smaller insert size than expected indicating a possible insertion. However, the limited read size with SRS is not sufficient enough to properly call the SV.

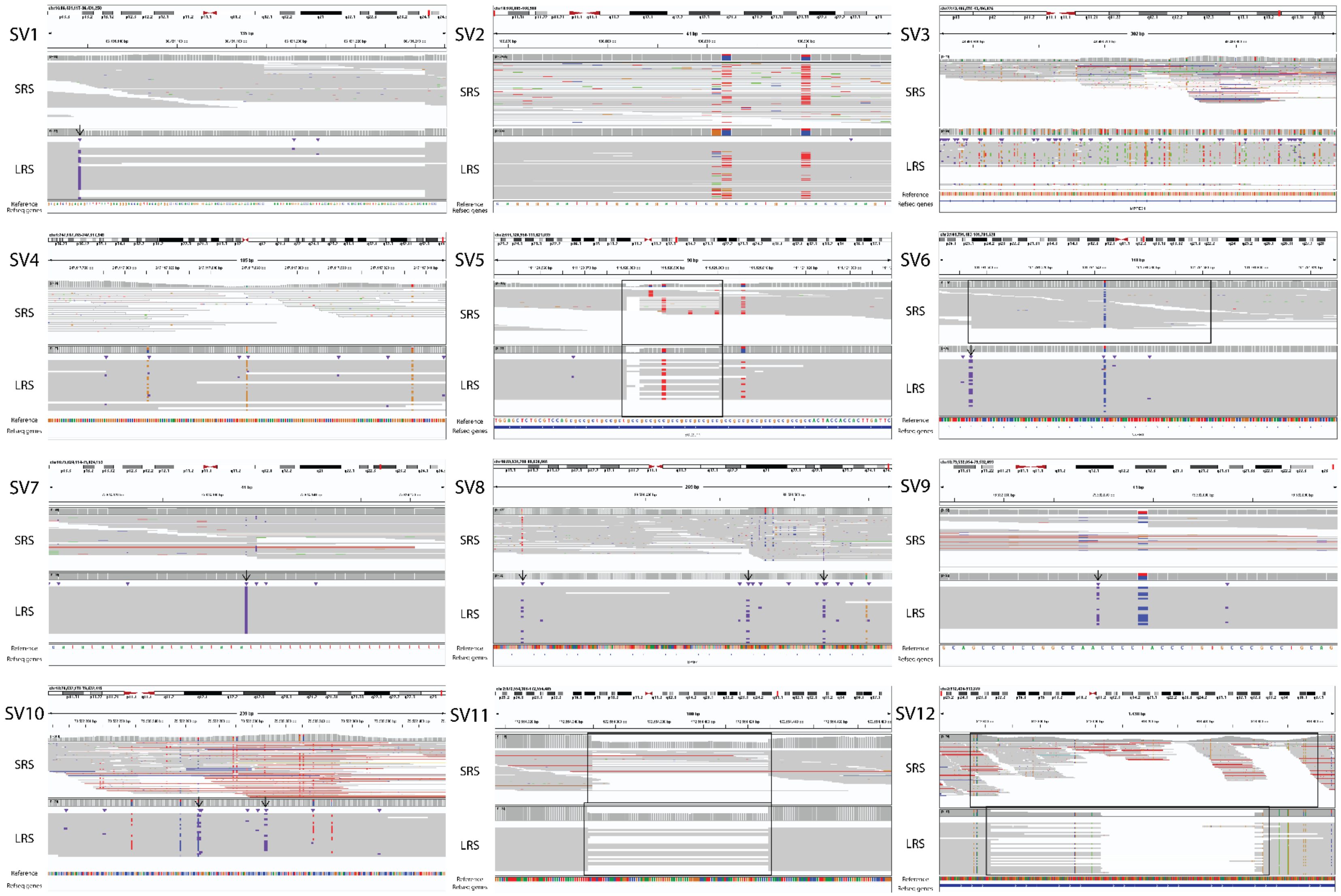

Fig. S5A:
Overview of the alignments of SV1-SV12 detected by LRS and SRS. For each SV this figure shows the SRS alignment in the top part and LRS alignment in the bottom part. Black arrows and boxes indicate where the SV is visible in the SRS and/or LRS alignments.

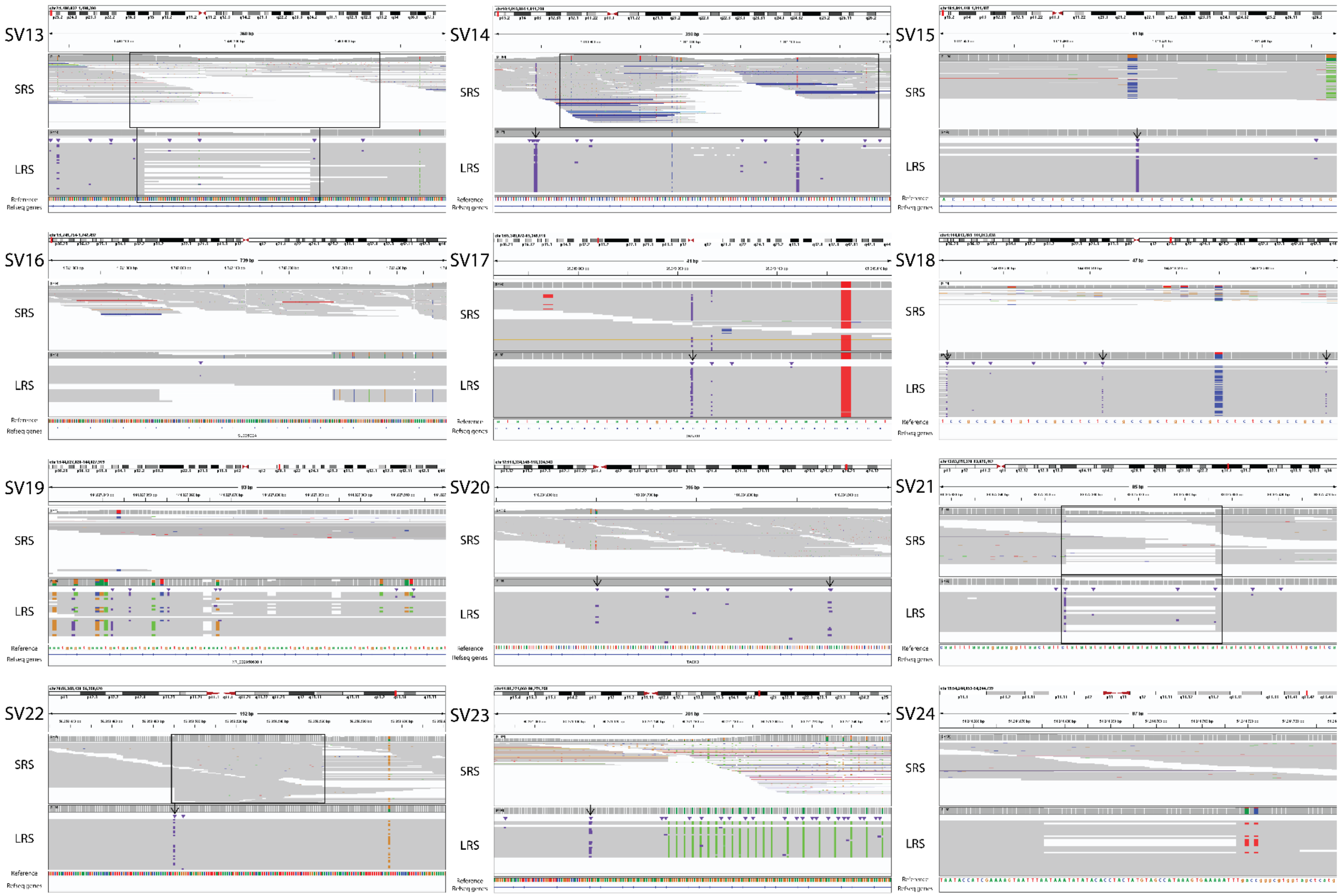

Fig. S5B:

Overview of the alignments of SV13-SV24 detected by LRS and SRS. For each SV this figure shows the SRS alignment in the top part and LRS alignment in the bottom part. Black arrows and boxes indicate where the SV is visible in the SRS and/or LRS alignments.

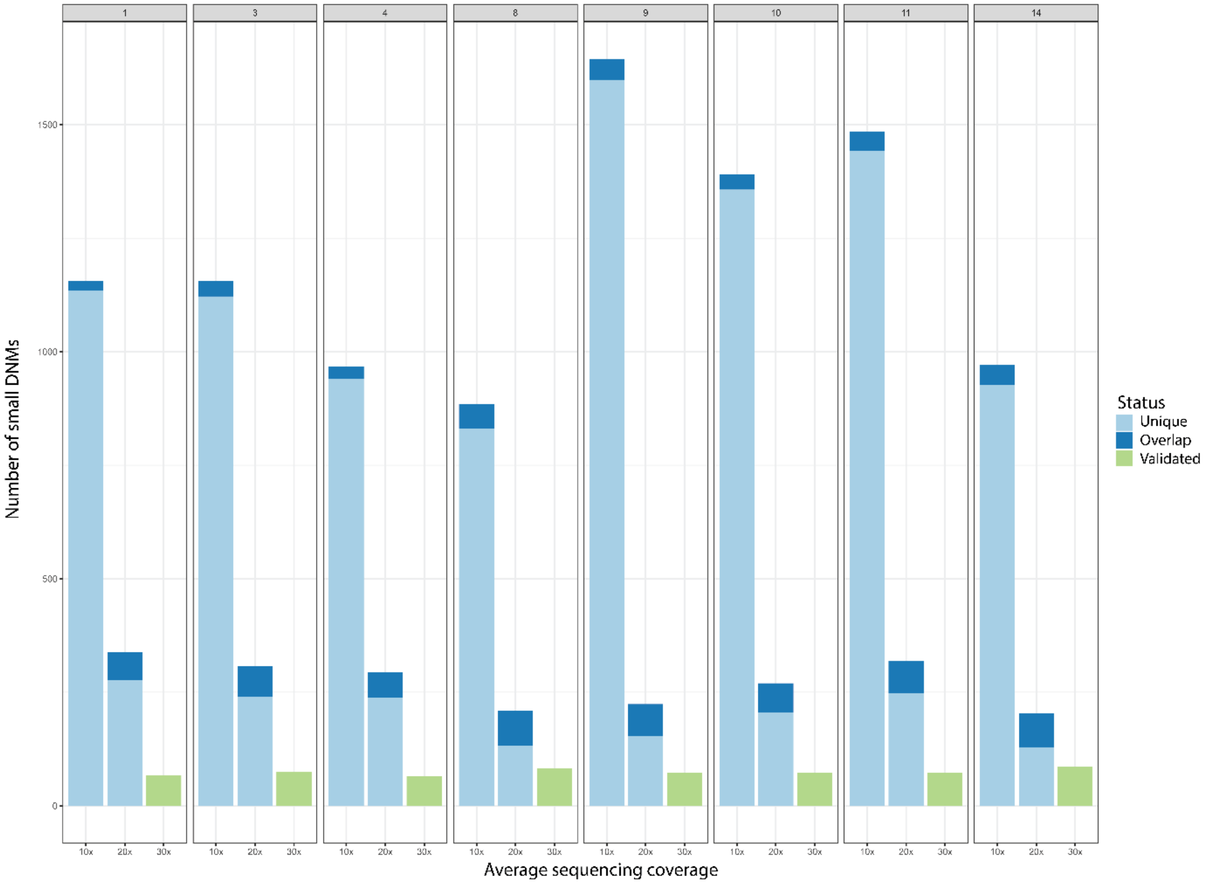

Fig. S6: Titration results

Effects of lower coverage on DNM detection for each trio. X-axis shows the coverage level, from average 30x (full) to 20x and 10x. Grey boxes indicate the trio identifier. Colors indicate the DNM status. ”Validated” denotes the DNMs that are either detected at the full coverage by both platforms or experimentally validated. Overlap denotes the DNMs that overlap with the validated set; “Unique” denotes the small DNMs that are detected only at that specific coverage.

Supplementary Tables

Table S1: Overview of sequenced trios

From left to right columns indicate the Trio ID, gender of the proband, birth year for the probands, types of microarray analysis that is run on the sample (if any) and number of SMRT cells used for the PacBio LRS run per sample.

Trio ID	Gender of Child	Year of birth	# SMRT Cells Child	# SMRT Cells Father	# SMRT Cells Mother
trio 1	Male	2003	3	3	3
trio 3	Male	2005	3	3	3
trio 4	Male	2014	5	6	6
trio 8	Male	2007	3	3	3
trio 9	Male	2016	5	3	3
trio 10	Female	2015	3	3	3
trio 11	Male	2004	3	3	3
trio 14	Male	2013	3	3	3

Table S2A: LRS statistics

From left to right columns indicate the Trio ID, sample name, average coverage of the aligned reads, average mapping quality of aligned reads, error rate of the aligned reads computed per base, percentage of GC bases in all aligned reads, total number of reads, average length of the reads, longest read length.

Trio	Sample	Average Coverage	Mapping Quality	Error Rate	GC %	Number of Reads	Average Read Length	Longest Read Length
1	Child	30.30	46.42	1.34 %	40.39%	9,847,810	17,103	49,994
1	Father	25.34	46.49	1.49 %	40.23%	4,680,942	16,420	49,818
1	Mother	25.46	46.48	1.45 %	40.58%	5,268,160	18,369	48,724
2	Child	29.23	46.89	1.44 %	40.55%	5,472,047	17,690	50,107
2	Father	26.68	46.22	1.51 %	40.16%	5,852,017	18,280	49,953
2	Mother	25.93	46.31	1.40 %	40.16%	5,706,913	17,881	47,550
3	Child	27.39	46.57	1.51%	40.32%	4,894,816	17,454	50,160
3	Father	26.77	46.48	1.50%	40.62%	5,785,153	17,079	49,690
3	Mother	29.27	46.77	1.53%	39.98%	4,536,886	17,605	49,447
4	Child	34.00	46.28	1.41%	40.50%	5,285,739	17,837	50,024
4	Father	54.10	46.43	1.43%	40.50%	4,398,973	17,921	47,007
4	Mother	41.50	46.34	1.38%	40.23%	4,801,356	16,509	49,598
5	Child	31.29	46.40	1.42%	40.52%	6,017,764	15,118	47,708

5	Father	28.15	46.60	1.55%	40.45%	4,828,973	17,193	53,900
5	Mother	38.50	46.59	1.52%	40.57%	5,032,396	16,024	48,280
6	Child	30.30	46.43	1.41%	40.66%	4,924,740	17,301	48,840
6	Father	24.69	46.44	1.13%	40.14%	4,963,483	16,782	49,921
6	Mother	31.09	46.52	1.53%	40.44%	5,794,226	15,707	42,962
7	Child	31.08	46.48	1.39%	40.36%	5,975,065	17,706	49,010
7	Father	34.36	46.69	1.22%	40.62%	7,644,820	16,887	49,953
7	Mother	32.79	46.41	1.33%	40.65%	5,576,438	17,468	49,204
8	Child	27.44	46.57	1.42%	40.59%	5,607,329	15,618	48,843
8	Father	31.74	46.73	1.35%	40.60%	7,346,751	16,310	48,625
8	Mother	25.67	46.69	1.23%	40.32%	5,486,194	17,187	50,174

Table S2B: **SRS statistics**
From left to right columns indicate the Trio ID, sample name, mean coverage of the aligned reads, mapping quality of the aligned reads, total number of reads, percentage of the mapped reads.

Trio	Sample	Mean Coverage	Mapping Quality	Error rate	GC %	Number of Reads (M)	Mapped read %
1	Child	54.9	31.1	0.97%	40.61%	1,220	99.37%
1	Father	65.7	31.1	0.98%	40.61%	1,452	99.53%
1	Mother	54.7	30.6	0.97%	40.56%	1,209	99.55%
3	Child	71.4	31.1	0.81%	40.79%	1,579	99.65%
3	Father	68.8	31.0	0.81%	40.82%	1,517	99.74%
3	Mother	77.9	30.6	0.81%	40.83%	1,719	99.68%
4	Child	81.8	30.9	0.87%	40.92%	1,827	99.43%
4	Father	80.7	31.1	0.85%	40.72%	1,797	99.53%
4	Mother	88.6	31.0	0.82%	40.86%	1,973	99.49%
8	Child	64.2	31.4	0.84%	40.88%	1,421	99.62%
8	Father	70.6	30.8	0.81%	40.76%	1,558	99.69%
8	Mother	75.6	31.4	0.81%	40.80%	1,673	99.63%
9	Child	90.0	31.8	0.82%	40.67%	1,985	99.80%
9	Father	103.4	31.7	0.80%	40.60%	2,287	99.78%
9	Mother	70.8	31.8	0.79%	40.65%	1,556	99.84%

10	Child	58.6	30.6	0.77%	40.61%	1,289	99.79%
10	Father	62.5	31.0	0.81%	40.45%	1,377	99.76%
10	Mother	50.4	31.1	0.80%	40.51%	1,107	99.76%
11	Child	69.5	30.6	0.76%	40.75%	1,527	99.81%
11	Father	92.0	31.1	0.76%	40.62%	2,029	99.80%
11	Mother	59.9	30.9	0.75%	40.72%	1,316	99.80%
14	Child	74.6	31.5	0.94%	40.91%	1,643	99.76%
14	Father	65.7	31.2	1.04%	40.95%	1,451	99.67%
14	Mother	81.5	31.4	0.97%	40.88%	1,797	99.72%

Table S3A: SNV Calling overview

From left to right columns indicate the Trio ID. From second to last rows indicate total number of called substitutions by LRS, total number of called substitutions by SRS, total number of shared substitutions between LRS and SRS, total number of called substitutions unique to LRS, total number of called substitutions unique to LRS in regions not covered by SRS, total number of called substitutions unique to SRS, total number of called substitutions unique to SRS in regions not covered by LRS, number of Mendelian inheritance errors in LRS called substitutions that are shared by SRS, number of Mendelian inheritance errors in SRS called substitutions that are shared by LRS, number of Mendelian inheritance errors in called substitutions unique to LRS, number of Mendelian inheritance errors in called substitutions unique to SRS.

**Trio**	**1**	**3**	**4**	**8**	**9**	**10**	**11**	**14**
Total Variants LRS	4,045,771	4,023,351	4,029,953	4,049,469	4,165,169	4,076,626	4,039,050	4,079,542
Total Variants SRS	4,039,069	4,040,763	4,049,922	4,048,968	4,207,325	4,068,711	4,046,122	4,091,258
Shared Variants	3,809,897	3,789,427	3,793,559	3,800,859	3,938,457	3,830,185	3,790,064	3,843,073
Unique Variants LRS	235,874 (5.8%)	233,924 (5.8%)	236,394 (5.9%)	248,610 (6.1%)	226,712 (5.4%)	246,441 (6.0%)	248,986 (6.2%)	236,469 (5.8%)
In No Coverage Regions	107,733 (2.7%)	106,459 (2.6%)	109,222 (2,7%)	118,050 (2.9%)	97,543 (2.3%)	115,942 (2.8%)	120,637 (3.0%)	109,527 (2.7%)
Unique Variants SRS	229,172 (5.7%)	251,336 (6.2%)	256,363 (6.3%)	248,109 (6.1%)	268,868 (6.4%)	238,526 (5.9%)	256,058 (6.3%)	248,185 (6.1%)
In No Coverage Regions	90,295 (2.2%)	105,703 (2.6%)	113,670 (2.8%)	105,086 (2.6%)	117,255 (2.8%)	97,242 (2.4%)	109,526 (2.7%)	107,115 (2.6%)
MIE Overlap LRS	1,567 (0.04%)	1,519 (0.04%)	1,550 (0.04%)	1,409 (0.04%)	1,588 (0.04%)	1,509 (0.04%)	1,864 (0.05%)	1,579 (0.04%)
MIE Overlap SRS	1,090 (0.03%)	962 (0.03%)	968 (0.03%)	1,097 (0.03%)	842 (0.02%)	1,007 (0.03%)	995 (0.03%)	1,004 (0.03%)
MIE Unique LRS	4,664 (2.0%)	5,027 (2.1%)	4,413 (1.9%)	4,983 (2.0%)	4,737 (2.1%)	5,108 (2.1%)	5,893 (2.4%)	4,808 (2.0%)
MIE Unique SRS	524 (0.2%)	606 (0.2%)	897 (0.2%)	596 (0.2%)	671 (0.2%)	568 (0.2%)	696 (0.3%)	657 (0.3%)

Table S3B: **Indel variant calling overview**

From left to right columns indicate the Trio ID. From second to last rows indicate total number of called indels by LRS, total number of called indels by SRS, total number of shared indels between LRS and SRS, total number of called indels unique to LRS, total number of called indels unique to LRS in regions not covered by SRS, total number of called indels unique to SRS, total number of called indels unique to SRS in regions not covered by LRS, number of Mendelian inheritance errors in LRS called indels that are shared by SRS, number of Mendelian inheritance errors in SRS called indels that are shared by LRS, number of Mendelian inheritance errors in called indels unique to LRS, number of Mendelian inheritance errors in called indels unique to SRS.

**Trio**	**1**	**3**	**4**	**8**	**9**	**10**	**11**	**14**
Total Variants LRS	1,000,831	1,007,910	994,271	993,683	1,012,085	1,009,160	1,002,073	1,006,835
Total Variants SRS	909,467	922,787	923,834	920,711	945,860	926,296	928,408	933,328
Shared Variants	582,158	582,413	579,927	581,269	593,085	588,180	585,104	590,551
Unique Variants LRS	418,673 (41.8%)	425,497 (42.2%)	414,344 (41.7%)	412,414 (41.5%)	419,000 (41.4%)	420,980 (41.7%)	416,969 (41.6%)	416,284 (41.3%)
In No Coverage Regions	117,507 (11.7%)	117,678 (11.7%)	116,521 (11.7%)	116,651 (11.7%)	114,763 (11.3%)	118,380 (11.7%)	117,090 (11.7%)	116,920 (11.6%)
Unique Variants SRS	327,309 (36.0%)	340,374 (36.9%)	343,907 (37.2%)	339,442 (36.9%)	352,775 (37.3%)	338,116 (36.5%)	343,304 (37.0%)	342,777 (36.7%)
In No Coverage Regions	89,751 (9.9%)	93,035 (10.1%)	94,111 (10.2%)	92,554 (10.1%)	95,058 (10.0%)	92,250 (10.0%)	93,570 (10.1%)	93,361 (10.0%)
MIE Overlap LRS	13,633 (2.3%)	15,256 (2.6%)	12,017 (2.1%)	11,199 (1.9%)	16,049 (2.7%)	8,027 (1.4%)	11,484 (2.0%)	12,107 (2.1%)
MIE Overlap SRS	19,191 (3.3%)	17,098 (2.9%)	15,164 (2.6%)	16,189 (2.8%)	17,316 (2.9%)	11,940 (2.0%)	16,923 (2.9%)	17,786 (3.0%)
MIE Unique LRS	38,936 (9.3%)	43,947 (10.3%)	35,862 (8.7%)	31,336 (7.6%)	46,029 (11.0%)	34,284 (8.1%)	33,247 (8.0%)	35,325 (8.5%)
MIE Unique SRS	50,211 (15.3%)	43,329 (12.7%)	40,739 (11.8%)	42,019 (12.4%)	42,986 (12.2%)	45,675 (13.5%)	43,045 (12.5%)	46,291 (13.5%)

Table S4A: Summary of the number of identified *de novo* substitution in LRS
From top to bottom rows indicate number of *de novo* substitutions, insertions, deletions, and the sum total of al *de novo* mutations.

	Trio1	Trio3	Trio4	Trio8	Trio9	Trio10	Trio11	Trio14	Sum	Mean
Substitutions	67	80	68	76	75	77	74	84	**601**	**75.1**
Insertions	3	8	3	4	3	2	4	1	**28**	**3.5**
Deletions	4	4	2	9	6	5	6	7	**43**	**5.4**
Total	74	92	73	89	84	84	84	92	**672**	**84**

Table S4B: **Summary of the number of identified *de novo* substitution in SRS**
From left to right rows indicate number of *de novo* substitutions, insertions, deletions, and the sum total of al *de novo* mutations by trio.

	Trio1	Trio3	Trio4	Trio8	Trio9	Trio10	Trio11	Trio14	Sum	Mean
Substitutions	89	127	82	99	107	80	89	90	**763**	**95.4**
Insertions	3	8	2	6	2	4	2	3	**30**	**3.8**
Deletions	6	6	7	11	7	11	6	12	**66**	**8.3**
Total	98	141	91	116	116	95	97	105	**859**	**107.4**

Table S5: **All small DNMs detected by LRS**

For all small DNMs detected by LRS this table provides the genomic position, chromosomal variant, coding variant, variant group, additional variant details, quality scores (AQ and GQ), GATK score and validation status. The column cluster shows whether the small DNM was considered a clustered DNM or not (see **Methods**).

External file: Additional file 2 - Table S5.xlsx

Table S6A: **Overlap between LRS and SRS small DNMs**

From top to bottom rows indicate the total number of small *de novo* mutations detected by LRS, the total number of small SRS-detected *de novo* mutations, number of small *de novo* mutations detected by both sequencing platforms, number of LRS-detected small *de novo* mutations that are detected by only one *de novo* caller in SRS (see **Methods**) and the number of small DNMs uniquely detected by LRS.

	**Trio1**	**Trio3**	**Trio4**	**Trio8**	**Trio9**	**Trio10**	**Trio11**	**Trio14**	**Sum**	**Mean**
Total LRS	74	92	73	89	84	84	84	92	**672**	**84**
Total SRS	98	141	91	116	116	95	97	105	**859**	**107.4**
Overlap	66	73	63	83	73	69	73	83	**583**	**72.9**
LRS+	3	6	6	4	3	5	3	5	**35**	**4.4**
LRS-unique	5	13	4	2	8	10	8	4	**54**	**6.8**

Table S6B: **Overlap between SRS and LRS small DNMs**

From top to bottom rows indicate the total number of LRS-detected small *de novo* mutations, total number of SRS-detected small *de novo* mutations, number of small *de novo* mutations detected by both sequencing platforms, number of SRS small DNMs that overlap with the LRS lenient set (see **Methods**) and the number of small DNMs uniquely detected by SRS.

	**Trio1**	**Trio3**	**Trio4**	**Trio8**	**Trio9**	**Trio10**	**Trio11**	**Trio14**	**Sum**	**Mean**
Total SRS	98	141	91	116	116	95	97	105	**859**	**107.4**
Total LRS	74	92	73	89	84	84	84	92	**672**	**84**
Overlap	66	73	63	83	73	69	73	83	**583**	**72.9**
SRS+	16	15	20	24	32	11	14	11	**143**	**17.9**
SRS-unique	16	53	8	9	11	15	10	11	**133**	**16.6**

Table S7: Coding small *de novo* mutations detected by LRS and SRS

For all coding small DNMs detected by LRS and SRS, this table provides the genomic position, chromosomal variant, coding variant, variant group, additional variant details, quality scores (AQ and GQ), GATK score and validation status.

External file: Additional file 3 - Table S7.xlsx

Table S8: **All small DNMs detected by SRS**

For all small DNMs detected by SRS this table provides the genomic position, chromosomal variant, coding variant, variant group, additional variant details, quality scores (AQ and GQ), GATK score and validation status.

External file: Additional file 4 - Table S8.xlsx

Table S9A: Validation overview LRS-unique small DNMs
From top to bottom rows indicate the variant type. From left to right columns indicate the total number of detected small DNMs, the number of small DNMs with successful primer design, the number of confirmed small DNMs, the percentage of confirmed small DNMs.

	Total	Primer design	Confirmed	% Confirmed
LRS-unique	54	27	11	20.3%
LRS-unique substitution	28	13	11	39.3%
LRS-unique insertion	17	10	0	0%
LRS-unique deletion	9	4	0	0%

Table S9B: Validation overview SRS-unique small DNMs
From top to bottom rows indicate the variant type. From left to right columns indicate the total number of detected small DNMs, the number of small DNMs with successful primer design, the number of confirmed small DNMs, the percentage of confirmed small DNMs.

	Total	Selected	Confirmed	% Confirmed
SRS-unique	133	42	8	6.0%
SRS-unique substitution	93	34	7	7.5%
SRS-unique insertion	12	2	0	0%
SRS-unique deletion	28	6	1	3.6%

Table S10: LRS-unique small DNMs

Overview of the LRS-unique small DNMs and the reasoning why these small DNMs were not detected by SRS. For each of the 11 DNMs this table shows the allele quality (AQ) reflecting the quality of the alternate allele, genotype quality (GQ) reflecting the quality of the genotype, but also whether the mutation is located in a repeated region of the genome, whether is well covered in both LRS and SRS, and whether it was called in SRS. Finally the table presents the reason why the DNM was not in the overlap set. Called potentially maternally or paternally inherited means that due to a small number of alternative reads in the respective parent, the mutation was not called *de novo* in SRS, but was marked potentially inherited. Low quality *de novo* mutation means that for one of the parents there was a small number of alternative reads at the position of the DNM.

Trio	Position (GRCh38)	AQ	GQ	Repeat region?	Well covered in LRS?	Well covered in SRS?	Called in SRS?	Reason why not in overlap
1	chr1:198079019-198079019	54	54	No	Yes	Yes	Yes	Called as potentially maternally inherited
3	chr16:52469597-52469597	56	56	No	Yes	Yes	Yes	Called as potentially maternally inherited
4	chr7:115997201-115997201	58	58	Yes	Yes	Yes	Yes	Called as potentially paternally inherited
4	chr11:46576054-46576054	57	58	No	Yes	Yes	Yes	Called as potentially paternally inherited
10	chr5:64759449-64759449	57	58	No	Yes	Yes	Yes	Called as potentially maternally inherited
10	chr6:97491419-97491419	60	60	Yes	Yes	Yes	Yes	Called as potentially maternally inherited
10	chr11:132965130-132965130	67	67	Yes	Yes	Yes	Yes	Called as potentially maternally inherited
10	chr15:37884087-37884087	52	52	Yes	Yes	Yes	Yes	Called as potentially maternally inherited
14	chr6:166597462-166597462	46	46	Yes	Yes	Yes	Yes	Called as potentially maternally inherited
14	chr10:131290026-131290026	45	31	No	Yes	Yes, but low mapping quality	Yes	Called as low quality *de novo* mutation
14	chr18:75551066-75551066	58	58	No	Yes	Yes	Yes	Called as potentially paternally inherited

Table S11: SRS-unique small DNMs

Overview of the SRS-unique small DNMs and the reasoning why these small DNMs were not detected by LRS. For each of the eight DNMs this table shows the GATK sequencing score and whether the DNM was located in a repeated region of the genome. Additionally, the table shows whether the DNM was well covered in both SRS and LRS. Finally the table indicates whether the DNM was called in LRS and the reason why it was not called (*de novo*) in LRS. The reasoning in the last column refers back to the *de novo* mutation filtering criteria we applied to the LRS data (see **Methods**).

**Trio**	**Position (GRCh38)**	**GATK score**	**Repeat region?**	**Well covered in SRS?**	**Well covered in LRS?**	**Called in LRS?**	**Reason why not called (*de novo*) in LRS**
1	chr1:166916439-166916439	769.77	No	Yes	Yes	Yes	Insufficient genotype quality for mother
1	chr5:39982266-39982266	1179.77	No	Yes	Yes	Yes	Alternative allele depth is 3 in father
1	chr20:62301762-62301762	995.77	Yes	Yes	Yes	Yes	Insufficient genotype quality for father
3	chr19:872379-872379	996.77	No	Yes	No	No	Insufficient coverage
4	chr10:43125792-43125792	1359.77	No	Yes	Yes	Yes	No genotype for father
4	chr18:10449641-10449641	1065.77	No	Yes	Yes	Yes	No genotype for mother
10	chr6:78918297-78918297	153.73	Yes	Yes	Yes	Yes	Alternative allele depth mother is 4 and variant allele frequency proband <20%
10	chr20:37038456-37038456	368.77	Yes	Yes	Yes	No	Alternative allele depth is 3 in proband

Table S12A: STRs in LRS data

Overview of the number of STR loci that have been genotyped for each sample. In addition, the number of completely genotyped STR loci are shown. Finally this table shows the number of repeat loci for which one or both alleles in the child was ≥2 repeat units longer or shorter than the number of repeat units in both parents and how many of these fall in an NDD gene.

Sample	STRs genotyped	STRs genotyped for entire trio	Number of STR loci with *de novo* length	Number of high quality candidate *de novo* STRs
child	171,101	171,043	119	4
father	171,082			
mother	171,095			
child	171,103	171,042	110	3
father	171,094			
mother	171,087			
child	171,031	170,979	115	6
father	171,087			
mother	171,105			
child	171,076	171,037	85	6
father	171,081			
mother	171,103			
child	171,104	171,049	86	1
father	171,103			
mother	171,101			
child	171,111	171,056	86	1
father	171,095			
mother	171,093			
child	171,099	171,055	141	1
father	171,099			
mother	171,101			
child	171,094	171,042	107	6
father	171,087			
mother	171,096			

Table S12B: STRs in SRS data

Overview of the number of STR loci that have been genotyped for each sample. In addition, the number of completely genotyped STR loci are shown. Finally this table shows the number of repeat loci for which one or both alleles in the child was ≥2 repeat units longer or shorter than the number of repeat units in both parents and how many of these fall in an NDD gene.

Sample	STRs genotyped	STRs genotyped for entire trio	Number of STR loci with *de novo* length	Number of high quality candidate *de novo* STRs
child	171,131	171,109	283	26
father	171,138			
mother	171,122			
child	171,137	171,122	249	19
father	171,137			
mother	171,134			
child	171,134	171,121	268	31
father	171,134			
mother	171,134			
child	171,121	171,109	268	12
father	171,122			
mother	171,132			
child	171,133	171,112	124	5
father	171,131			
mother	171,129			
child	171,135	171,112	158	9
father	171,131			
mother	171,123			
child	171,130	171,109	247	14
father	171,137			
mother	171,125			
child	171,126	171,113	225	10
father	171,135			
mother	171,113			

Table S13: Overview of all high quality *de novo* STRs in LRS

From left to right columns indicate trio number, genomic start position of the STR, variant group, number of repeat units in GRCh38/Hg38 reference genome, number of repeat units of both allele for patient, father and mother, TRGT quality score of both alleles for patient, father and mother and finally the validation result.

External file: Additional file 5 - Table S13.xlsx

Table S14: Overview of all high quality *de novo* STRs in SRS

From left to right columns indicate trio number, genomic start position of the STR, variant group, number of repeat units in GRCh38/Hg38 reference genome, number of repeat units of both alleles for patient, father and mother, ExpansionHunter confidence intervals for both alleles of the patient and finally the validation result.

External file: Additional file 6 - Table S14.xlsx

Table S15: Overlap between LRS and SRS for *de novo* STRs

From top to bottom rows indicate by which technologies the STR was called. If STRs were called by both technologies these are listed in the bottom row. The columns indicate the trio in which the STR was detected as well as the sum of all called *de novo* STRs and the mean.

	**Trio1**	**Trio3**	**Trio4**	**Trio8**	**Trio9**	**Trio10**	**Trio11**	**Trio14**	**Sum**	**Mean**
LRS	4	3	6	6	1	1	1	6	**28**	**3.5**
SRS	26	19	31	12	5	9	14	10	**126**	**15.8**
Overlap	0	1	0	0	0	0	0	0	**1**	**0.1**

Table S16: Validation overview for LRS-unique and SRS-unique *de novo* STRs

Rows indicate to which of the two technologies the unique variants belong. The columns indicate the total number of high-quality *de novo* STR expansions and contractions, the number of selected STRs and the validation results in false positives, inherited variants and confirmed variants.

	Total	Attempted	False positive	Inherited	Confirmed	% Confirmed
LRS-unique	27	18	14	4	0	0%
SRS-unique	125	18	13	5	0	0%

Table S17: Overview of SVs in LRS and SRS

From left to right columns indicate Trio ID, genomic position in HGVS format, structural variant type, length of the structural variant, genic region that structural variant is located (if any). *indicates the SV that was detected by both LRS and SRS.

External file: Additional file 7 - Table S17.xlsx

Table S18: **Effect of coverage on DNM detection**
From left to right columns indicate Trio ID, number of *de novo* mutations detected at 10x, number of *de novo* mutations that overlap between 10x and the truth set, percentage of small DNMs that overlap between 10x and truth set, number of *de novo* mutations detected at 20x, number of *de novo* mutations that overlap between 20x and truth set, percentage of small DNMs that overlap between 20x and the truth set.

**Trio**	**10x**	**10x Overlap**	**10x Sensitivity %**	**20x**	**20x Overlap**	**20x Sensitivity %**	**Truth set**
1	1158	22	32.84%	340	61	91.04%	67
3	1163	34	45.33%	319	67	89.33%	74
4	969	27	41.54%	300	56	86.15%	65
8	888	54	65.06%	213	77	92.77%	83
9	1648	46	63.01%	228	71	97.26%	73
10	1395	33	45.21%	274	64	87.67%	73
11	1486	43	58.90%	322	71	97.26%	73
14	973	44	51.16%	210	75	87.21%	86

Table S19A: **Phasing of LRS detected DNMs and parental age**

From left to right columns indicate the Trio ID, total number of *de novo* mutations in the proband, number of phased *de novo* mutations by Whatshap, number of *de novo* mutations phased as paternal, number of *de novo* mutations phased as maternal, percentage of the phased *de novo* mutations, paternal age of the trio, maternal age of the trio.

Trio	# small DNMs	Phased DNMs	Paternal	Maternal	% Phased	Paternal / Maternal Age
1	74	72 (97%)	52	20	97.30	31 / 38
3	92	86 (94%)	64	22	93.48	43 / 41
4	73	70 (96%)	53	17	95.89	32 / 26
8	89	85 (96%)	69	16	95.51	36 / 37
9	84	80 (95%)	50	30	95.25	37 / 39
10	84	81 (96%)	60	21	96.43	35 / 29
11	84	80 (95%)	63	17	95.24	51 / 43
14	92	88 (96%)	75	13	95.65	34 / 24
**Total**	**672**	**642 (96%)**	**486**	**156**	**95.53**	**NA**

Table S19B: **Phasing of SRS detected DNMs**

From left to right columns indicate the Trio ID, total number of *de novo* mutations in the proband, number of phased *de novo* mutations by Whatshap, number of *de novo* mutations phased as paternal, number of *de novo* mutations phased as maternal, number of *de novo* mutations failed to be phased parentally, percentage of *de novo* mutations that are phased parentally.

Trio	# small DNMS	Phased DNMs	Paternal	Maternal	Unknown	% phased	Paternal / Maternal Age
1	98	50 (51%)	18	8	24	26.53%	31 / 38
3	141	67 (48%)	20	4	43	17.02%	43 / 41
4	91	48 (53%)	17	4	27	23.07%	32 / 26
8	116	45 (39%)	17	5	23	18.96%	36 / 37
9	116	58 (50%)	25	8	25	28.44%	37 / 39
10	95	34 (36%)	10	5	19	15.78%	35 / 29
11	97	44 (45%)	11	1	32	12.37%	51 / 43
14	105	41 (39%)	16	2	23	17.14%	34 / 24
**Total**	**859**	**387 (45%)**	**134**	**37**	**216**	**20.02%**	**NA**

Table S20: **Phasing details of small DNMs detected by both LRS and SRS**
For all small DNMs that were phased by both LRS and SRS this table provides the genomic position, additional variant details and phasing details including the genotype, haplotype, and paternal and maternal counts for both LRS and SRS.

External file: Additional file 8 - Table S20.xlsx

Table S21: Phasing details of small DNMs uniquely detected by LRS

For small DNMs that were detected uniquely by both LRS, this table provides the genomic position, validation and phasing status.

Trio	Position	Validation Status	Phasing Status
1	chr1:198079019-198079019	True *de novo*	Maternal
3	chr16:52469597-52469597	True *de novo*	Maternal
4	chr7:115997201-115997201	True *de novo*	Paternal
4	chr11:46576054-46576054	True *de novo*	Paternal
10	chr5:64759449-64759449	True *de novo*	Maternal
10	chr6:97491419-97491419	True *de novo*	Maternal
10	chr11:132965130-132965130	True *de novo*	Maternal
10	chr15:37884087-37884087	True *de novo*	Maternal
14	chr6:166597462-166597462	True *de novo*	Maternal
14	chr10:131290026-131290026	True *de novo*	Paternal
14	chr18:75551066-75551066	True *de novo*	Paternal
3	chr4:121944414-121944414	No variant	Maternal
3	chr5:114373640-114373640	No variant	Unknown
3	chr7:125225466-125225466	Inherited	Unknown
3	chr11:26235002-26235002	Inherited	Unknown
4	chr2:134443265-134443265	Inherited	Unknown
4	chr2:216636925-216636925	No variant	Unknown
8	chr2:22739501-22739501	Inherited	Unknown
8	chr12:93478936-93478938	Inherited	Unknown
9	chr3:29779706-29779706	Inherited	Unknown
10	chr1:60735436-60735440	Inherited	Unknown
11	chr1:239304289-239304289	No variant	Maternal
11	chr2:81167641-81167641	Inherited	Unknown
11	chr5:85362386-85362386	No variant	Paternal
11	chr7:157478544-157478544	Inherited	Unknown
11	chr11:115288462-115288463	Inherited	Unknown
11	chr17:3965846-3965846	Inherited	Unknown
